# Supplementary material for: The efficacy and safety of lignocaine-embedded dissolvable microneedle versus EMLA for topical analgesia in adults undergoing venepuncture: A single-centre, parallel-group, double-blind randomised clinical trial protocol in a tertiary care setting
Source: PLoS One. 2025 Nov 4;20(11):e0335932. doi: 10.1371/journal.pone.0335932 (PMC12585024; doi:10.1371/journal.pone.0335932)
Supplement: S2 Table — (DOCX) [file pone.0335932.s002.docx]

| **Table 2. Components of the WHO Registration Data Set. Extracted from the ClinicalTrials.gov registry and adapted from the SPIRIT 2025 guideline.** | |
| --- | --- |
| **Components** | **Details** |
| **Primary registry and trial identifying number** | Clinicaltrials.gov registry  NCT05694858  Weblink: https://clinicaltrials.gov/study/NCT05694858 |
| **Date of registration in the ClinicalTrials.gov registry** | 19/01/2023 |
| **Secondary Identifying Number** | UKM/PPI/111/8/JEP-2022-736  MOSTI.D (S) 600-4/19/15  FF-2022-401 |
| **Universal Trial Number (UTN)** | N/A |
| **Source of monetary and material support** | Malaysian Technology Development Fund 2 (TED2) Grant (Grant number: MOSTI.D (S) 600-4/19/15)  Faculty of Medicine Grant, Universiti Kebangsaan Malaysia (UKM) (Grant number: FF-2022-401) |
| **Primary Sponsor** | Faculty of Medicine,  Hospital Canselor Tuanku Muhriz (HCTM),  Universiti Kebangsaan Malaysia (UKM),  Jalan Yaacob Latif, Bandar Tun Razak,  56000, Cheras,  Kuala Lumpur, Malaysia.  Email: [sepukm@ukm.edu.my](mailto:sepukm@ukm.edu.my) |
| **Secondary Sponsor** | N/A |
| **Contact for Public Queries** | Primary contact: Dr Muhammad Irfan Abdul Jalal  Email: [irfan.abduljalal@ukm.edu.my](mailto:irfan.abduljalal@ukm.edu.my)  Study contact backup: Dr Lam Chen Shen  Email: [lamchenshen@ukm.edu.my](mailto:lamchenshen@ukm.edu.my) |
| **Contact for Scientific Queries** | Professor Dr. Fook-Choe Cheah  Email: [cheahfc@hctm.ukm.edu.my](mailto:cheahfc@hctm.ukm.edu.my) |
| **Public Title** | Transdermal Microneedle Lignocaine Delivery Versus EMLA Patch for Topical Analgesia Before Venepuncture Procedure To Adults in Clinical Setting |
| **Scientific Title** | Transdermal Microneedle Lignocaine Delivery Versus EMLA Patch for Topical Analgesia Before Venepuncture Procedure To Adults in Clinical Setting |
| **Countries of Recruitment** | Malaysia |
| **Health Condition** | Ophthalmology Patients (Glaucoma, Cataract and other ophthalmological conditions) Attending Ophthalmology Clinic at HCTM Requiring Intravenous Cannulation |
| **Interventions** | **Phase I:** Single group trial. Lignocaine-embedded microneedle array patch (LEMAP)  **Phase II:**  a) **Experimental Arm:** LEMAP  A biodegradable maltose microarray needle (MAN) patch loaded with 12.5 mg lignocaine will be applied on the dorsum of the participant's hand for 30 minutes. Intravenous cannulation will be carried out after 30 minutes.  **Control arm:** Eutectic Mixture of Local Anaesthetics **(**EMLA) patch containing lidocaine and prilocaine in a 1:1 ratio  One finger-tip unit (1 FTU) of EMLA containing 12.5 mg lignocaine and 12.5 mg prilocaine will be applied on the dorsum aspect of the participant's hand. This will then be covered by a Polyvinyl Alcohol (PVA)-Polyethylene Terephthalate (PET) adhesive and left in place for 30 minutes. Intravenous cannulation will be carried out after 30 minutes. |
| **Key Eligibility Criteria** | For both Phase I and II  **Inclusion Criteria:**  1) Patients aged 18 years old and above  2) Patients requiring venous cannulation for blood investigations before ophthalmological procedures at the outpatient department.  **Exclusion Criteria:**  1) Patient with a previous history of sensitization or allergy to lignocaine.  2) Patient with a previous history of allergy to materials used in the study i.e., plaster, electrodes, maltose, Polyvinyl Alcohol (PVA), and Polyethylene Terephthalate (PET)  3) Patient exposed to analgesic usage within 24 hours prior to the procedure  4) Generalized skin disorder/ rash  5) Agitated/fretful/uncooperative patient  6) Uncommunicative/deaf/mute patients  7) Patients on hypnotics, or chronic pain relief medications  8) Patients with psychiatric conditions  9) Patients with hepatic impairment  10) Patients who are on CYP450 3A4, 3A5 or 1A2-inducing or inhibiting drugs (erythromycin, ciprofloxacin, amiodarone etc.) or pharmacotherapeutic agents that affect hepatic blood flow (metoprolol) since both may affect the metabolism of lignocaine  11) Failed first/single attempt at venepuncture after the application of LEMAP or PET patch for the control arm. |
| **Trial Type** | **Purpose:** Treatment  **Allocation:** Open Label (Non-randomized, single group trial) (Phase I); Randomized Controlled Trial (Phase II)  **Framework**: Superiority (Phase II)  **Masking / Blinding**: Unblinded (Phase I); Single Blinded [Outcome assessors  **Assignment:** Parallel (Phase II)  **Type of endpoint**: Pharmacokinetics profile of lignocaine (Phase I); Efficacy (Phase II)  **Phase**: I and II |
| **Date of First Enrolment** | 01/06/2025 |
| **Sample Size** | **Phase I:** 20 participants  **Phase II:** 72 participants per LEMAP and EMLA group (n_total_ = 144 participants; including 10% attrition rate) |
| **Recruitment Status** | **Phase I**  Completed  **Phase II**  Recruiting |
| **Primary Outcome** | **Phase II**  1) **Visual Analogue Score (VAS):** VAS score is measured in a continuous scale (range 0-100). It is obtained using a Med-05-100 VAS Pain Scale ruler (Schlenker Enterprises Ltd, Lombard, USA) with 0-100 mm slider. It is measured based on the pain experienced on the IV cannulated hand. Higher VAS score indicates greater intensity or degree of pain whilst lower VAS score indicates lesser pain intensity.  The measurements will be made at 1 minute after IV cannulation which will be inserted following lignocaine-impregnated microneedle patch or standard EMLA patch application  2) **Skin Conductance Algesimeter Index:** The skin conductance peaks per second, measured in microSiemens per second (μS/s), is obtained using PainMonitor™ (Med-Storm Innovation AS, Oslo, Norway) device on the hypothenar eminence of the opposite hand not receiving IV cannulation. Higher skin conductance algesimeter index indicates greater pain intensity and lower values indicate lesser pain intensity.  The measurements will be made at 1 minute after IV cannulation, which will be inserted following lignocaine-impregnated microneedle patch or standard EMLA patch application. |
| **Secondary Outcomes** | Nil |
| **Ethics Review** | **Status:** Approved  **Approval Date:** 12/12/2022  **Approval ID:** UKM/PPI/111/8/JEP-2022-736,  **Contact:** UKM Research Ethics Committee, 1^st^ Floor, Clinical Block, Hospital Canselor Tuanku Muhriz, Jalan Yaacob Latif, Bandar Tun Razak, 56000, Cheras, Kuala Lumpur (**Contact number:** +603-91455046/5048) |
| **Data Sharing Statement** | Anonymized and de-identified participants’ clinical and trial outcome data will be shared through the Harvard Dataverse Repository (<https://dataverse.harvard.edu/>). The full statistical codes used for trial data cleaning, transformation and analysis will be made available on the GitHub repository. |
| N/A: Not Available; WHO: World Health Organization; LEMAP: Lignocaine-Embedded Microneedle Array Patch; EMLA: Eutectic Mixture of Local Anaesthetics (containing lidocaine and prilocaine in a 1:1 ratio) | |
