# Supplementary material for: The efficacy and safety of lignocaine-embedded dissolvable microneedle versus EMLA for topical analgesia in adults undergoing venepuncture: A single-centre, parallel-group, double-blind randomised clinical trial protocol in a tertiary care setting
Source: PLoS One. 2025 Nov 4;20(11):e0335932. doi: 10.1371/journal.pone.0335932 (PMC12585024; doi:10.1371/journal.pone.0335932)
Supplement: S1 File — (PDF) [file pone.0335932.s003.pdf]

Research Protocol

Transdermal microneedle lignocaine delivery versus EMLA patch for topical analgesia before venepuncture procedure to adults in a clinic setting

Date of Document: 12th December 2023 (version 3.0)-*revised*

PRINCIPAL INVESTIGATOR: Professor Dr Cheah Fook Choe

CO-INVESTIGATORS: Professor Dr Azrul Azlan Hamzah

Professor Dr Mae-Lynn Catherine Bastion

Dr Muhammad Irfan Abdul Jalal

Dr Lam Chen Shen

Ms. Chua Xin Yun

COLLABORATORS: Dr Goh Chee Seong

CHAPTER 1

INTRODUCTION

1.1 Transdermal Drug Delivery (TDD)

Venepuncture is one of the most commonly encountered invasive medical procedures that causes significant traumatic pain experience and stress to the hospitalised patients. The anxiety and apprehension toward needle puncture may be exacerbated in patients with chronic conditions who require frequent venous cannulation or venepuncture. This in long term may affect their emotional well-being and eventually deter them from seeking future medical attention.

With the current advances in medical science and technology, healthcare is continually improving with innovative approach to holistically deliver treatment to the patients efficiently and effectively. Local anaesthesia prior to venepuncture is regarded as a modern approach that fits with the Good Clinical Practice (GCP) framework in providing a quality standard of care to the hospitalised patients. More recently, the emergence of transdermal drug delivery system (TDDS) has been valued for its invention as a less invasive alternative in facilitating effective drug administration.

The conventional drug-delivery route through hypodermic needle injection and topical cream are the most common strategy for systemic drug delivery through the skin surface. However, both methods are having their inherent downsides. The greatest challenge associated with hypodermic needle use is the pain, stress, and even needle phobia caused by its invasiveness which leads to lower patient compliance and acceptability.[1,2] On the other hand, the topical drug delivery with formulated cream (e.g. lignocaine cream) would be a more attractive alternative route of administration since it is painless and can be self-administered by the patients themselves. Nonetheless, as the drugs must passively diffuse across the skin, its onset of action and efficacy might be affected by its limited bioavailability at the site of action.[3] Such delayed or substandard drug efficacy would thus be impractical and unsuitable for widespread and convenient use in the modern-day fast-paced clinical setting.

Besides, previous researches have investigated various state-of-the-art TDDS alternatives such as iontophoresis (induction of electrical current to drive electrostatic diffusion of charged anaesthetic permeants through the skin layers), sonophoresis (generation of low-frequency ultrasound that facilitates the penetration of drugs), electroporation (formation of microchannels upon electric pulse to increase permeability), magnetophoresis (utilisation of magnetic field), thermophoresis (short thermal exposure to ablate the skin surface for better diffusion of drugs), and the jet injection (controlled compression of gas or spring to deliver anaesthetic drug into targeted skin layer).[4–6] However, these myriad strategies of enhancing transdermal drug delivery are hampered by several design pitfalls and undesirable adverse reactions such as second-degree burns, cross-contamination risk, limited absorption for drugs of large molecular size and others.[1] Hence, a more revolutionary approach is required to circumvent the issues above.

1.2 Skin Structure and Transdermal Drug Delivery (TDD)

Human skin is the most readily accessible and largest organ in the human body which covers and area of 1.5 - 2.0m^2 and accounts for 16% of the total body weight of an adult.[1,7] The primary

role of human skin is to serve as a barrier that protects the human body against the relatively hostile external environment. However, such organised barrier function has also led to a poor permeation of the applied topical drug across the skin layer, hence impairing effective drug delivery and onset of action.

The skin is histologically classified into 3 main compartments: (1) the outermost epidermis, (2) the middle dermal layer, and (3) the innermost hypodermis.[1,8,9] The avascular layer of epidermis predominantly consists of keratinocytes (~95% of total cell in epidermis) which are the non-viable constituents of the outermost *stratum corneum* (SC) and the viable cutaneous sublayers underneath the SC. Several sublayers of the viable epidermis, i.e. *stratum basale, stratum spinosum, stratum granulosum,* and *stratum lucidum* are collectively 50-100 µm thick and are adjoint together by tonofibrils. On the whole, the whole skin epidermal layer is 50-150 µm thick.[10]

The epidermis is partitioned from the deeper dermal layer by an undulating epidermal-dermal junction. Dermis is the thickest substructure of the skin (2-3 mm thick) and it is primarily composed with collagen and elastin fibres that confer it the strength and elasticity.[11] The papillary and reticular layers of the dermis house the blood vessels, nerves, lymphatic vessels, skin appendages, and the connective tissues.[8,9] Lying underneath it is the innermost hypodermis or the subcutaneous layer, which is an elastic layer that is mainly constituted of the adipose tissues, blood vessels and nerve endings.[9] **Figure 1** illustrated the structure and composition of the human skin.

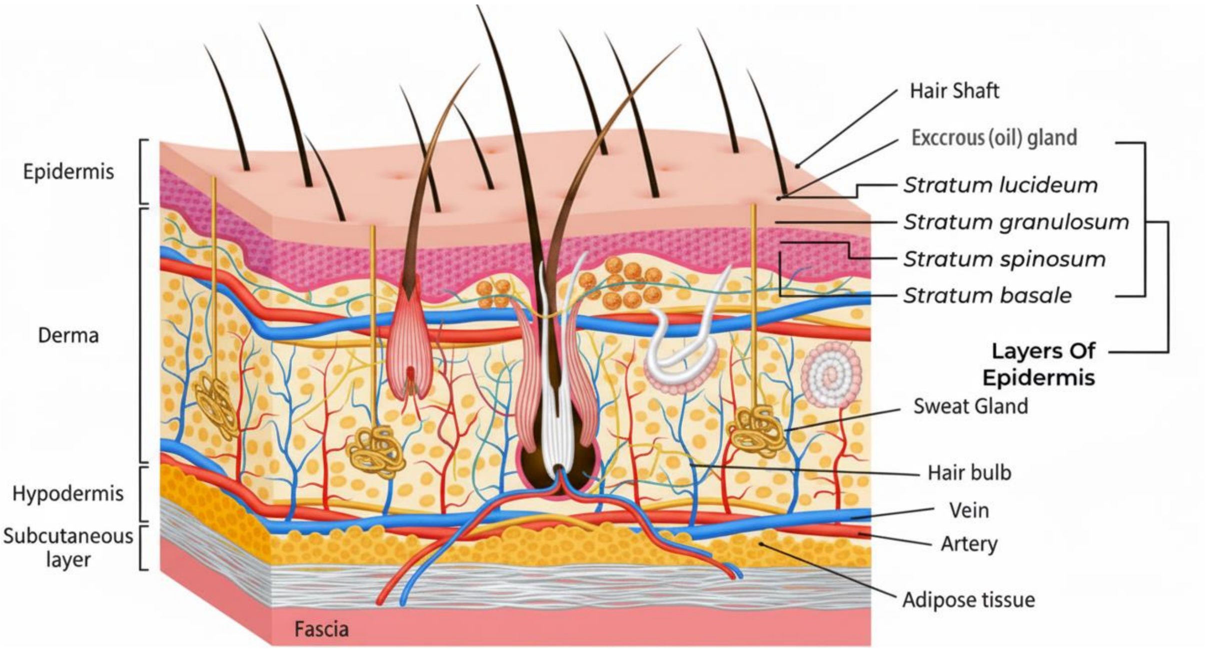

Figure 1. Human skin structure and composition. The skin is primarily composed of three layers: epidermis, dermis, and fascia, with distinct structural elements located in specific anatomical positions. (Author's own image, created by Biorender)

In the context of TDD, the *stratum corneum* (SC) is the primary mechanical barrier that limits the uptake rate of the topically applied drugs. The SC, made up of multilayered dead corneocytes scattered throughout a lipid-rich matrix, is principally a lipophilic nature by its barrier properties and which will exhibit a selective permeability for the penetration of relatively lipophilic molecules into deeper layers of the skin.[12] Therefore, drugs that are rather lipophobic or high in molecular weight are relatively impeded for its effective systemic delivery. Apart from that, the underlying epidermal-dermal junction would be the secondary factor conferring the resistance on the molecular transportation of drug molecules across the epidermal layer into dermis.[13] Only the drug molecules that managed to get through the SC and the papillary dermis will effectively reach the systemic circulation to exert their systemic effects.[14]

The sophisticated structure of the skin has also been implicated as the primary barrier to ensuring an optimal bioavailability of the drugs delivered using TDDS. Previous study reported that only 10-20% the total drug in a topical preparation successfully permeates across the skin layers.[15] On the contrary, the high bioavailability of the drugs (90-100% of the total drug) has been touted as the main advantage of the hypodermic needle.[16] However, its invasive and pain-inducing nature again limits its widespread usage.[16] Hence, a novel TDD system is required to overcome the aforementioned design limitations.

CHAPTER 2
LITERATURE REVIEW

2.1 Emergence of Microneedle and its Applications

With the advent of the microfabrication technology, a revolutionary solution of effective transdermal drug delivery has been introduced with the use of microneedle (MN) patch. MN is a medical device that is composed of tiny micron-sized needles aligned in out-of-plane protruded arrays that can be impregnated with bioactive drug substances.[17] These micron-sized needles create multiple transdermal microchannels when they puncture through the cutaneous stratum corneum. Consequently, this innovative means of drug delivery enhances transcutaneous drug absorption, resulting in a faster onset of therapeutic action.[16]

MN offers several superior clinically-relevant benefits. The most significant advantage of MN is that it minimises the pain experienced by the patients by preventing nociceptor stimulation during venepuncture or intravenous cannulation. Further, the MN design could also be tailored for the intended therapeutic agent delivery.[16] Besides, MN can also be readily self-administered by the patients without the expert assistance from the healthcare professionals.[16,17] With regard to needle breakage issue associated with the conventional hypodermic needle usage, the dissolvable MN is potentially attractive in circumventing such a design imperfection. Furthermore, dissolving MN would also minimise the biohazard wastage compared to the conventional metal-based hypodermic needles.[18]

Maltose, a natural non-cytotoxic carbohydrate is one of the most common constituents utilised in MN fabrication due to its excellent biodegradability profile and quick dissolution within minutes of administration.[19,20] With a well-recognised safety record, maltose is also widely used for various pharmaceutical formulations with its proven safety and efficacy track record upon clinical application.[21] Among MN types, dissolvable MN is a type of biodegradable microneedle patch that can be encapsulated with pharmaceuticals for transdermal drug delivery. Dissolving drug-embedded microneedle patches allow a continuous dissolution of drugs into the targeted skin layer upon their insertion onto the skin surface.[22] Such a drug administration strategy could also be described as a ‘poke-and-release’ drug delivery system.[22]

To date, the scientific endeavours investigating the effects of dissolving microneedles on delivering anaesthetics for the adult patients who require regular blood transfusion by their clinical conditions are scarce. So far, only three prior studies evaluated the efficacy of microneedles for alleviating pain in adult patients undergoing routine peripheral venous cannulation. Rzhevskiy et al. (2022) demonstrated in a clinical trial investigating the efficacy of lidocaine delivered via a hollow microneedle (MicronJet600) prior to peripheral venous cannulation. They showed a significant 11-fold VAS score reduction in adults undergoing routine peripheral venous cannulation when 2% lidocaine was intradermally administered using MicronJet600 compared to no anaesthetic pretreatment (mean VAS score: 3.6 (MJ+L combination) vs 39.7 (placebo); Cohen’s d: -.43 (95% CI -48, -3.9)).[23] In addition, Ornelas et al. (2016) conducted a randomised, single-blinded, parallel-group clinical trial to evaluate the effect of microneedle pre-treatment in hastening the onset of cutaneously-applied 4% lidocaine cream. They demonstrated that the microneedle-assisted 4% lidocaine delivery had shortened the application time from 60 to 30 minutes and significantly minimised the pain induced by

needle lancet at 30 mins compared to sham patch (VAS score (mean ± SD): microneedle: 4 ± 1.3 mm; sham: 14.4 ± 3.8 mm).[24] In contrast, Gupta et al. (2012) compared the efficacy of lidocaine injections administered by hollow borosilicate-glass microneedle with the conventional hypodermic needle in 15 healthy adults. They established that both lidocaine delivery systems produce similar local anaesthetic effects ($p > 0.05$) across all time points, but the hollow microneedle recipients reported significantly better dermal analgesic effects compared to the recipients of conventional hypodermic needles.[25]

Based on these findings, the utility of microneedles is promising for effective anaesthetic delivery and pain amelioration. However, the validity of the aforementioned evidence may be limited by several methodological shortcomings. For instance, Ornelas et al's RCT was limited to only the male population, a significant methodological weakness that hampers the generalisability of the results to the whole population[24]. Apart from that, Gupta et al. (2012) stated that the hollow borosilicate-glass microneedle prototype suffers from undesirable lidocaine leakage when it was applied on their subjects.[25] Therefore, further investigations are necessary to improve the microneedle design so that the local anaesthetic delivery can be further optimised for convenient application in adults who undergo frequent routine clinical procedures such as peripheral venous cannulation or venepuncture.

To our knowledge, the efficacy of lignocaine-impregnated microneedle for local anaesthetic delivery has not been studied. Hence, the safety, tolerability and pharmacodynamic property of lignocaine-embedded microneedle warrant further investigation. Therefore, our trial is designed to provide the answers to such scientific lacunae.

2.2 The general pharmacological properties of lignocaine

Lignocaine, in its solid state, possesses a crystalline and colourless structure that is dissolvable into a hydrochloric salt form.[26] It possesses anti-arrhythmic and anti-nociceptive properties mediated through voltage-gated sodium and potassium channel blockade.[27]

It is primarily bound to α-acid glycoprotein (AAG) (approximately 50%) and to lesser extent, albumin (around 25%).[28,29] Diseases such as myocardial infarction and cirrhosis and other factors, for instance smoking and age, will influence the serum AAG concentration levels which in turn will affect the fraction of free plasma lignocaine concentration.[30-33] By contrast, an increase in the AAG level will reduce the amount of unbound, active lignocaine in the plasma, resulting in a reduction of its pharmacological effects at a specific plasma lignocaine concentration.[34] The volume of distribution at steady state, V_{ss}, in normal adults is 1.32 (SD 0.27) L/kg whilst the V_{ss} in chronic heart failure (CHF), chronic liver impairment and chronic renal failure (CRF) patients are 0.88 L/kg, 2.31 L/kg and 1.2 L/kg, respectively.[35,36]

Lignocaine has a high degree of hepatic extraction ratio (62%-81%).[37] Hence, factors affecting hepatic blood flow such as metoprolol-mediated reduction in hepatic blood flow, congestive heart failure and acute myocardial infarction will influence the biotransformation rate of lignocaine.[38,39] Apart from that, lignocaine is metabolized by CYP3A4, CYP3A5 and CYP1A2 through consecutive deethylation steps.[40,41] Therefore, concurrent administration of CYP1A2 and CYP3A4 inhibitors such as amiodarone, fluvoxamine, erythromycin and others causes mild-to-major elevations of serum lignocaine concentrations.[42-44] Lignocaine dosage reduction by up to 60% is thus warranted when a CYP1A2 inhibitor (e.g. fluvoxamine) is concomitantly administered with lignocaine.[45]

Lignocaine has two major metabolites; monoethylglycinexylidide (MEGX) and glycinexylidide (GX).[46] MEGX is responsible for both lignocaine's therapeutic and toxic effects whilst GX primarily causes lignocaine-associated toxicity.[47,48] Lignocaine is mainly eliminated via hepatic metabolism and the clearance rate ranges from 0.72 (SD 0.15) L/hr/kg in healthy adults[35] to 0.25 L/hr/kg in patients with Child-Pugh class C hepatic impairment.[49]

The main adverse events (AEs) associated with toxic levels of serum lignocaine affect two major organ systems; the central nervous system (CNS) and cardiovascular system (CVS). In the former, the major AEs are confusion, slurring of speech, paraesthesia around the lips and tongue, diplopia, tremor, seizures whilst sinus bradycardia, sinus arrest and disturbances in atrioventricular conduction are the major AEs in the latter.[50] The toxic effects of lignocaine will appear when serum lignocaine concentration exceeds 5 mg/L and convulsion occurs when it exceeds 10 mg/L.[51] Despite being associated with the development of nasal adenomas and tumours in murine models, lignocaine is not associated with cancer development in humans.[51-52]

In 1999, the US Food and Drug Administration (FDA) has approved the lidocaine 5% patch (Lidoderm®, Endo Pharmaceuticals Inc, Malvern, USA) for the treatment of postherpetic neuralgia.[53] Henceforth, it has been extensively investigated for the treatment of other medical conditions such as low back pain, postoperative pain control, rib fractures and for the prevention of venepuncture or injection-related pain in paediatric patients.[54-57] However, in a recent meta-analysis by Bai et al., the 5% topical lidocaine patch was ineffective as an adjunct for the management of acute and postoperative pain since no significant differences were found in terms of the mean pain intensity scores, duration of hospital stay and the postoperative opioid consumption between the 5% topical lidocaine patch and placebo recipients.[58] Hence, a new transdermal drug delivery system that can transcutaneously deliver lignocaine more effectively is required, especially in the context of alleviating pain associated with routine vein-puncturing procedures, namely intravenous cannulation and venepuncture.

2.3 Dermato-pharmacokinetics (DPK) and mathematical modelling of microneedle-assisted topical anaesthetic delivery

The dermal bioavailability of transdermally-delivered local anaesthetic agents should be accurately quantified in order to precisely evaluate their pharmacodynamic interactions with the cutaneous nociceptors. However, the quantification procedure is fraught with significant methodological challenges. Recently, Pensado et al. demonstrated that the minimally invasive stratum corneum (SC) sampling was only adequately-powered to detect a 50% difference in the mean uptake and clearance parameters and pharmacodynamic response characteristics (measured using the area above the blanching effect curve (AAEC)) between two different

doses of Betnovate® (0.1% w/w BMV) cream applied on the anterior aspects of forearms of 12 individuals, whilst a larger sample size was required to detect a smaller effect size (20% difference) for the two applied doses.[59] In contrast, the skin blanching technique produced more highly variable results and therefore was relatively indiscriminative to the two different Betnovate® cream doses.[59] Other techniques that have been proposed for dermato-pharmacokinetic (DPK) assessments of topical drugs, such as the in vitro Franz Diffusion Cell, ex vivo tape stripping, in-vivo micro-dialysis and suction blister techniques and confocal laser microscopy are also laden with serious methodological shortcomings (i.e. invasive procedure, absence of sensitive methods to analyze the analyte's concentration, technical variations in the tape removal).[60] Consequently, the DPK properties of topically-delivered local anaesthetics could not be accurately evaluated, which is the principal reason why our research will focus more on the TDD system's safety profile evaluation through the quantification of the amount of lignocaine or 5% EMLA dermal patch entering systemic circulation (classical bioavailability).

For an infinite dose of a given local anaesthetic agent, the quantity of penetrant or permeant that has reached the receptor chamber at time t, Q(t), can be represented by the following mathematical relationship:[61]

$$Q(t) = A * p * h * c* \left[D * \frac{t}{h^2} - \frac{1}{6} - \frac{2}{\Pi^2} \sum_{n=1}^{\infty} \frac{(-1)^n}{n^2} * e^{\left(\frac{-D * n^2 * \Pi^2 * t}{h^2}\right)} \right] \quad \text{(Equation 2.1)}$$

where A is the skin's surface area that is available for drug molecule diffusion; p is the coefficient of partition between the donor vehicle and the homogenous membrane; h is the thickness of the membrane (path length traversed by the drug molecules); C is the permeant's concentration in the donor solution; * is the multiplication symbol and D is the coefficient of diffusion for the permeant in the membrane. From equation 2.1, it can be clearly observed that the delivery of active drug molecules is proportionally related to the skin's surface area. As a result, a topically-applied local anaesthetic must be correctly applied to the intended cutaneous surface area to ensure optimal absorption and delivery of the anaesthetic drug molecules to the target site action site (i.e. skin nociceptors).

From equation 2.1, we can also deduce that decreasing h will also increase Q(t). Since microneedles puncture through the skin's SC layer, this reduces h and thus increases Q(t). We, therefore, hypothesised that the increased quantity of local anaesthetic agents (penetrant) at the skin nociceptors (receptor chamber) will also enhance the analgesic property of the local anaesthetic agents, resulting in a faster onset of action and subsequent greater pain reduction. Our postulate is further corroborated by Ronnander et al. who demonstrated that a change in the dermal concentration of sumatriptan delivered via dissolvable polyvinylpyrrolidone (PVP) microneedle with respect to time can be mathematically modelled using:[62,63]

$$\frac{dc}{dt} = -(K_L c) + 4\left(\frac{k_D \tan\theta}{\rho * \cos\theta}\right) h^2 \left[\frac{\beta\rho - c}{v_0 + v_{c,0} - v_c}\right] \left[c_S - \left(\frac{1-\beta}{\beta}\right) c\right], \quad \text{(Equation 2.2)}$$

where K_L is the constant of elimination; c is the drug concentration in the skin layer, k_D is the constant for the rate of dissolution of a drug matrix (in cm/h), ρ is the drug matrix's density (or the microneedle's density (in g/cm^3)) , θ is the half angle (in degree) at the dissolvable microneedle's apex (or microneedle's height-length ratio), h is the height of the dissolvable

microneedle, β is the fraction of drug mass in the microneedle, $v_0 + v_{c,0} - v_c$ is the sum of the initial volume of the skin layer (cm^3) and the initial volume of the microneedle (cm^3) minus the volume of microneedle at the time when c is measured (cm^3) and c_s is the solubility of matrix polymer in a solvent (e.g. water, measured in g/cm^3). Using the Fick's second law of diffusion, equation 2.2 could be rewritten as[63,64]:

$$\frac{\delta c}{\delta t} = D\frac{\delta^2 c}{\delta y^2} + 4(\frac{k_D tan\,\theta}{\rho * cos\,\theta})h^2 \left[\frac{\beta\rho - c}{v_0 + v_{c,0} - v_c}\right]\left[c_s - \left(\frac{1-\beta}{\beta}\right)c\right], \qquad \text{(Equation 2.3)}$$

where D is the diffusion coefficient or diffusivity (cm^2/h) and $\frac{\delta^2 c}{\delta y^2}$ is the curvature of the concentration profile at a specific point (x-position) in space. The first term on the right-hand side of equation 2.2 denotes the diffusion of the drug across the cutaneous layers and the second term represents the estimate of drug released from its encapsulating polymer via dissolution.[62]

From equations 2.2 and 2.3, we could again conjecture that the concentration of a drug in the skin is dependent upon the drug loading (β), and the height of the dissolvable microneedle (h).[62-65] By increasing the height of the microneedle, the stratum corneum layer can be bypassed and the local anaesthetics can hence be rapidly delivered to the intended site of action (skin nociceptors). Therefore, we believe a lignocaine-embedded microneedle patch design may enhance the lignocaine's onset of action via a similar mechanism.

2.3 Justifications of study

Based on our brief review of literature, we have then pointed out three problem statements in bridging the knowledge gaps from previous research with the highlighted significance of our current study.

I) The safety profile of transdermally-delivered lignocaine that is directly embedded within the matrix of dissolvable microneedle in the adult patients requiring venepuncture during routine clinical settings have not been robustly and exhaustively tested in previous studies. This study aims to evaluate the safety and tolerability of lignocaine-embedded microneedle in this cohort of patients. The microneedle patch was designed and used (without impregnated drugs) in a previous project approved by this committee among paediatric thalassaemia patients. This trial protocol has been published in clinicaltrials.org and also in the Journal of Clinical Medicine.[82] The safety profile was good with side effects showing that out of 19 patients studied, none reported any serious adverse reactions (SARs) or sudden unexpected serious adverse reactions (SUSARs) to the microneedle, with only one case complained of mild itchiness at the patch site.

II) The pharmacokinetic properties of lignocaine that is directly delivered transdermally through embedment within dissolvable microneedle's matrix have not been investigated in the previous study. Therefore, a subset of this study patients will provide the preliminary pharmacokinetic data for lignocaine delivered via such administration route which will indicate the extent of absorption of lignocaine, if any at all, into the systemic circulation. Animal study in rats showed no scarring and complete healing after microneedle patching, and blood plasma lidocaine was subtherapeutic level, below 0.4ng/mL and diminished to almost nil after 150 minutes.

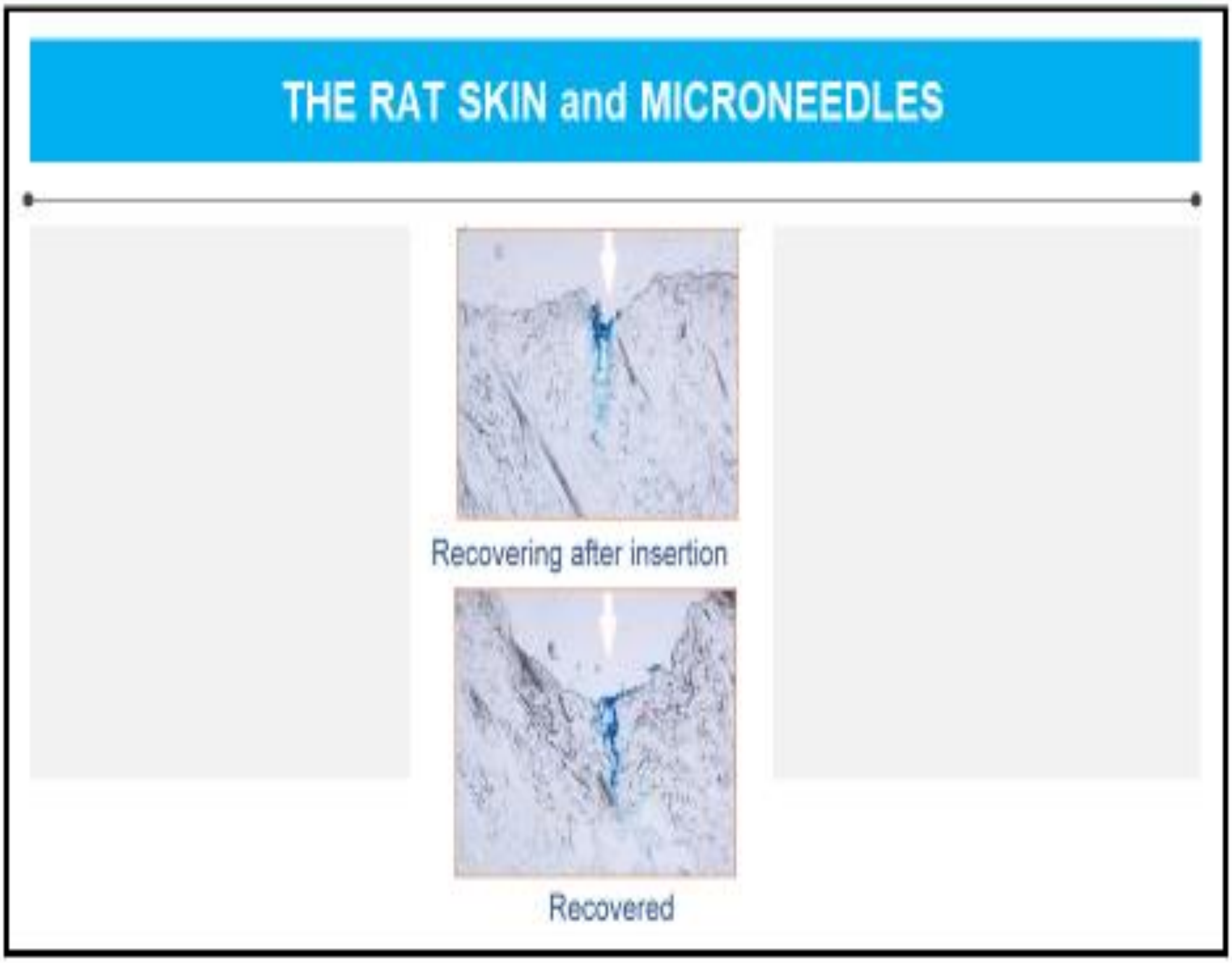

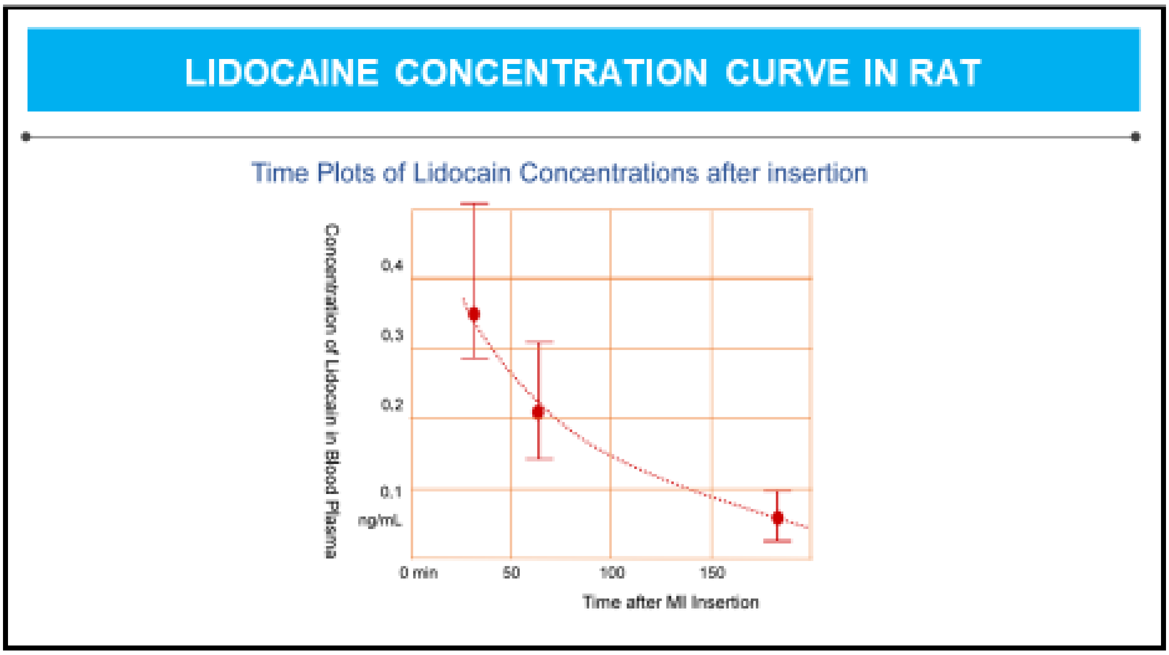

Hence, our current research is looking forward to providing the answers in response to the problem statements above as well as to highlight the potential of lignocaine-impregnated

microneedle for its clinical application in topical anaesthetic administration and management. To summarize the foundations of our research, the conceptual framework of this research is shown in Section 2.4.

2.4 Conceptual Framework

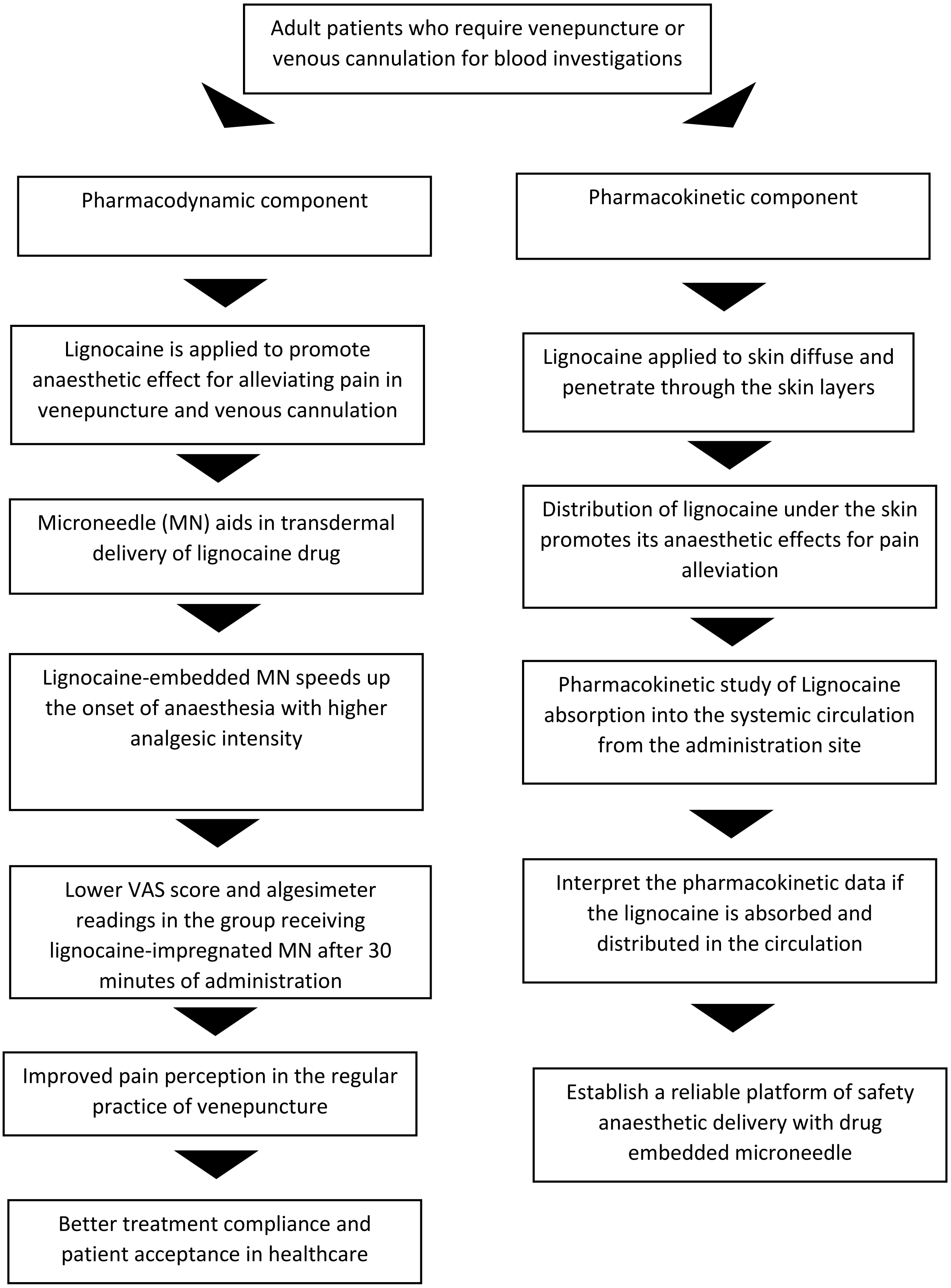

CHAPTER 3

OBJECTIVES

3.1 General Objective

To assess the safety and efficacy of lignocaine-embedded microneedles as a means of pain-reduction in adult patients requiring routine venepuncture procedure.

3.2 Specific Objectives

1. To evaluate the safety profile of lignocaine-embedded microneedle patch as a means of pain reduction in adult patients requiring routine vein-puncturing procedures

2. To assess the pharmacokinetic (PK) parameters of lignocaine in the systemic circulation when the transdermal lignocaine delivery is enhanced through microneedle usage.

3. To compare the efficacy of lignocaine-embedded microneedle patch with standard 5% EMLA dermal patch for pain reduction during venepuncture procedure based on mean changes in VAS scores and skin algesimeter index (pharmacodynamic (PD) study).

3.3 Research Questions

1. What is the safety profile of lignocaine-embedded microneedle patch used for pain reduction in adult patients requiring routine vein-puncturing procedures?

2. What are the values of PK parameters (AUC_{inf}, AUC_t, C_{max}, $t_{1/2}$, volume of distributions (V_d) and clearance) for the lignocaine constituent in adult patients receiving lignocaine-embedded microneedles for routine vein-puncturing procedures?

3. Are there differences between lignocaine-embedded microneedle patch and standard 5% EMLA dermal patch for pain reduction during venepuncture procedure based on mean changes in VAS scores and skin algesimeter index?

3.4 Research Hypotheses

1. H_0: The proportion of participants experiencing adverse event is not significantly different from 0.

   H_1: The proportion of participants experiencing adverse event is not significantly different from 0.

2. H_0: The values of PK parameters for the lignocaine constituent in adult patients receiving lignocaine-embedded microneedles are not bioequivalent with the values of PK parameters for the lignocaine constituent in adult participants receiving lignocaine through the standard topical route.

   H_1: The values of PK parameters for the lignocaine constituent in adult patients receiving lignocaine-embedded microneedles are bioequivalent with the values of PK parameters for the lignocaine constituent in adult participants receiving lignocaine through the standard topical route.

3. H_0: There are no differences in mean changes in VAS scores and skin algesimeter index between the trial participants receiving lignocaine-embedded microneedle patch and standard 5% EMLA dermal patch.

   H_1: There are significant differences in mean changes in VAS scores and skin algesimeter index between the trial participants receiving lignocaine-embedded microneedle patch and standard 5% EMLA dermal patch.

CHAPTER 4

METHODOLOGY

4.1 Study Design

This study can be divided into two distinct stages:

a) Stage 1: Non-randomized single-centre open-label single group clinical trial to primarily assess the safety and tolerability of lignocaine-impregnated microneedle in adult patients undergoing routine vein-puncturing related procedures (pharmacokinetic (PK) study).

b) Stage 2: A randomized single centre double blind two parallel group active controlled clinical trial to assess the efficacy of lignocaine-impregnated microneedle compared to 5% EMLA dermal patch (Pharmacodynamic (PD) study).

4.2 Study Location

The study will be carried out at the Ophthalmology Outpatient Clinic, Hospital Canselor Tuanku Muhriz (HCTM), UKM, Bandar Tun Razak.

4.3 Study Period

1st December 2022 – 30th November 2024 (24 months)

4.4 Eligibility Criteria

The inclusion and exclusion criteria of the study participants are as follows:

4.4.1 Inclusion Criteria

I) Patients aged 18 years old and above

II) Patients requiring venous cannulation for blood investigations before eye surgery

4.4.2 Exclusion Criteria

I) Patient with a previous history of sensitization or allergy to lignocaine.

II) Patient with a previous history of allergy to materials used in the study i.e., plaster, electrodes, maltose, Polyvinyl Alcohol (PVA), and Polyethylene Terephthalate (PET)

III) Patient exposed to analgesic usage within 24 hours prior to the procedure

IV) Generalized skin disorder/ rash

V) Agitated/ fretful / uncooperative patient

VI) Uncommunicative/deaf/mute

VII) Patients on hypnotics, or chronic pain relief medications

VIII) Patients with psychiatric conditions or cognitive impairment

IX) Patients with hepatic impairment

X) Patients who are on CYP450 3A4, 3A5 or 1A2-inducing or inhibiting drugs (erythromycin, ciprofloxacin, amiodarone etc.) or pharmacotherapeutic agents that affect hepatic blood flow (metoprolol) since both may affect the metabolism of lignocaine.

XI) Failed first/single attempt at venepuncture after the application of the MN patch

4.5 Reference Population

All adult patients undergoing vein-puncturing procedures at the Ophthalmology Outpatient Clinic in Malaysia.

4.6 Source Population

All adult patients undergoing vein-puncturing procedures (intravenous cannulation, venepuncture) at the Ophthalmology Outpatient Clinic, Hospital Canselor Tuanku Muhriz (HCTM), UKM, Bandar Tun Razak.

4.7 Sampling Frame

All adult patients undergoing vein-puncturing procedures (intravenous cannulation, venepuncture) at the Ophthalmology Outpatient Clinic, Hospital Canselor Tuanku Muhriz (HCTM), UKM, Bandar Tun Razak and fulfil the eligibility criteria.

4.8 Study Subjects

All adult patients undergoing vein-puncturing procedures (intravenous cannulation, venepuncture) at the Ophthalmology Outpatient Clinic, Hospital Canselor Tuanku Muhriz (HCTM), UKM, Bandar Tun Razak, fulfil the eligibility criteria and consent to trial participation.

4.9 Sampling Method

Stratified random sampling, a type of probability sampling method, will be used for both stages of the trial since the sample size required (section 4.9) is smaller than the whole pool of participants. Since differential pain experience has been shown in females and male adults[66], the stratification variable is the gender of the participants to ensure that our study sample contains an equal number of female and male adults and is representative of patient population.

4.10 Sample Size Calculation

4.10.1 Pharmacokinetic (PK) study

Due to the paucity of prior information, formal sample size calculation for stage 1 (pharmacokinetic (PK)) study based on power analysis cannot be carried out. Based on recommendations by Ogungbenro and Aarons (2010) and Julious (2012), the sample size is set at 20 subjects each for the PK study[67,68]. Even though it is recommended that the sample size can be minimally set at 12 subjects for a single-group pilot pharmacodynamic trial (Julious, 2012), the sample size is increased to 20 subjects since based on Julious 2012's results (Figure 3 of Julious 2012), the statistical asymptote is reached when the sample size is at least 20 subjects[68]. Hence, the addition of another subject will result in only non-substantial gain in the precision of the parameter estimates when the sample size of 20 participants is reached.

4.10.2 Pharmacodynamic (PD) Study

For stage II of the trial, the sample size was calculated using Power and Sample Size (PS) Program version 3.1.6 (Vanderbilt University, Nashville, Tennessee, USA; 2018). The standard deviation (SD) of the VAS score of 2.1 was obtained from a prior study and this is for patients who received 5% EMLA.[69] We consider a 1-point VAS difference between the intervention group as the minimum detectable difference (MTD) and type I error and study power (1- β) were fixed at 0.05 and 0.80, respectively. The ratio of controls to cases is fixed at a 1:1 ratio. Based on these parameter values, the calculated sample size is 70 participants per group.

After accounting for a 10% dropout rate, the final sample size is 77 participants per group (n_{total}= 154 participants) for this stage of trial.

4.11 Physical Description of the Lignocaine-Embedded Microneedle

Currently, we are able to manually produce some microneedle array patches (MAP) in a lab-scale quantity. Different designed parameters MAP have been used for initial testing on animal skin and also human skin. In this project, two major parts will be carried out. First, we will design a semi-automatic machine to fabricate MAP. Refined parameters will be adapted from previous designs for the fabrication of the drug-impregnated microneedles. Second, we will carry out a clinical trial for testing on the real implementation and application of the fabricated MAP. The details for each session are discussed below

Part I: Preparation of Biodegradable Lignocaine-Impregnated Microneedle Array Patch

In a previous project, a few parameters such as base plate size, number of microneedles per unit area, height, etc., have been determined and optimised. They will be used as a guideline for the design of new microneedle fabrication machine for better microneedle fabrication. With a new fabrication machine, we will be able to have a small-scale production to achieve a pre-commercialization technology readiness.

In this part, we will focus on the fabrication and implementation of a biodegradable microneedle array patch. Sugar compounds such as sucrose, trehalose, and maltose have been experimented with as biodegradable matrix materials for microneedles. In particular, maltose itself is a carbohydrate that is widely-acknowledged as a generally recognized safe excipient material for drug delivery. MAP fabricated from maltose generally demonstrates strong mechanical properties, which can facilitate the perforation of skin and the formation of microchannels for transdermal drug delivery. Additionally, maltose enables the function as a dissolvable MAP, allowing it to rapidly dissolve in the dermal regions within minutes at body temperature. This property enables the delivery of therapeutic compounds, such as proteins and peptides, in a rapid, safe, and environmentally friendly manner. The MAP that we propose as a prototype will consist of two basic but essential structures, i.e. the microneedles and the substrate (baseplate). This special design allows the microneedles to be separated from the substrate once the MAP is applied onto the skin. While the substrates can be readily disposed of after being peeled off from the skin, the microneedles and the therapeutic loads they carry would stay on the skin and continue to dissolve and release drugs in a predetermined and sustained manner. Furthermore, as the microneedle is only 150 µm in length, the maximum penetration would only reach the epidermis-dermis intersection. There is no possibility of the microneedles reaching the blood vessels, due to their limited length. Therefore, the drug distribution will follow a topical mode of distribution. No systemic distribution of the drug is expected.

A. Preparation of microneedle matrix mix for the dissolving microneedle

I. To prepare the calcium ion cross-linked alginate/sugar (Ca2+/Alg-sugar) composites, sodium alginate powder is first dissolved in DI water at a weight ratio of 1:4 with stirring in a water bath at 60 °C until a homogeneous solution is gained.

II. And then, the 15% (w/w) $CaCl_2$ solution is added slowly with rapid mixing to cross-link alginate (CaCl2/Cross-linked alginate weight ratio = 1:10).

III. To enhance the mechanical properties of composite microneedles, 15% (w/w) maltose monohydrate or any carbohydrate equivalents is added simultaneously into the sodium alginate solution to form a precursor for the preparation of a paste for the fabrication of drug-loaded microneedles and baseplate (substrate):

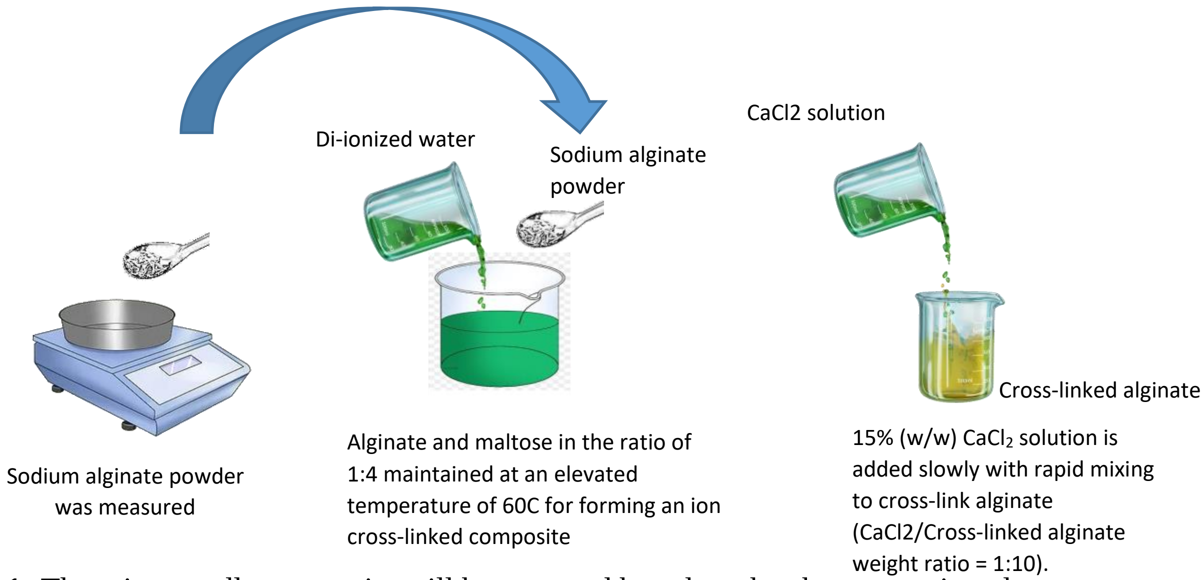

Figure 1: The microneedle composite will be prepared based on the above-mentioned steps (Authors' own image)

B. Fabrication of a mould as a microneedle stamp used to fabricate negative mould with polydimethylsiloxane (PDMS) via the moulding technique

A mould will be fabricated based on this fabrication process. The moulds for different sizes, shapes and physical scales of microneedles will be designed.

I. In the previous lab-scale fabrication, the pyramidal microneedle patch will be fabricated using polymethyl methacrylate (PMMA) and used as the male mould. In this project, we will use more solid and hard materials such as metal for the male mould.
II. Subsequently, reversed mould (which is the female mould for the microneedle fabrication) is fabricated. To do so, a PDMS mixture is blended and added to a dust-free master structure, followed by centrifugation (4000 rpm, 30 min, 25°C, centrifuged and application of vacuum (200 mbar, 30 min, 25°C, vacuum drying oven)) to remove air bubbles.
III. After curing at 100°C for 5 h, the female mould is obtained, which can be used to produce microneedles made of sugar compounds (such as maltose).
IV. The above processes are to prepare a reverse mould with different physical parameters to test for the suitability and conformity of the mould for fixing onto the semi-auto fabrication machine. After that, the designs have the highest aptness will be used. The parameters will be adapted for use with a 3D printer to facilitate the small-scale production of these moulds.

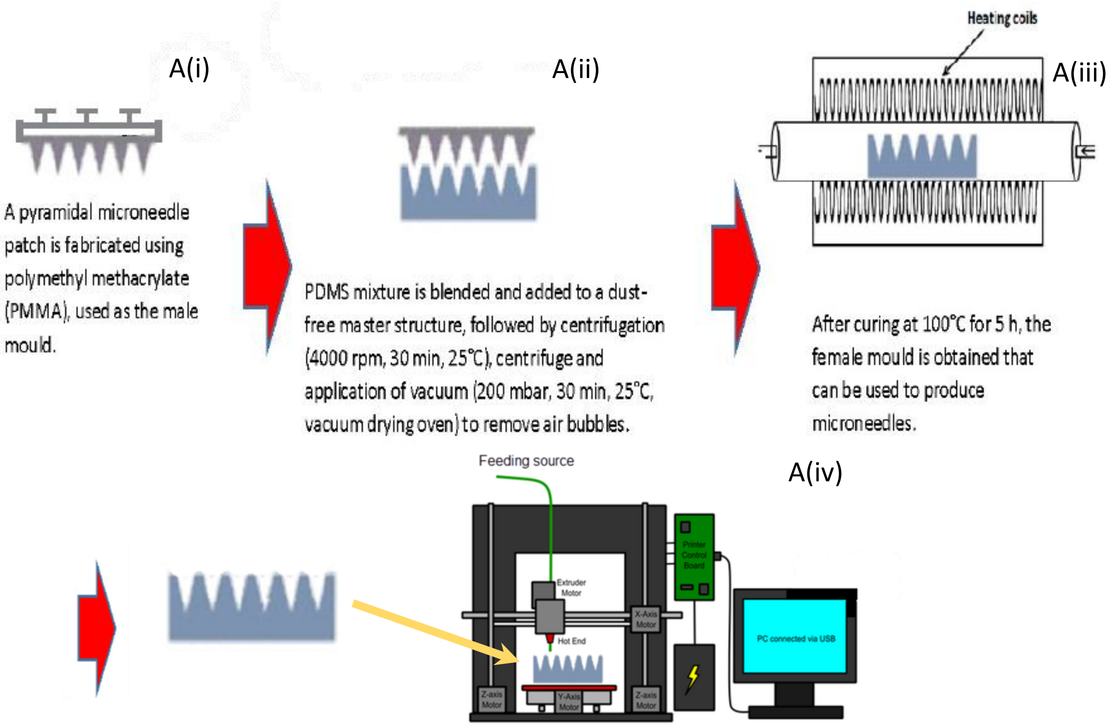

Female mould with the best parameters will be used as standard for small –scale production of the mould with different design and physical parameters used for microneedle fabrication

Figure 2: Moulds with different sizes, shapes and physical parameters will be designed and prepared (Authors' own image)

C. **Semi-auto machine for the fabication of dissolving microneedle array patch**

With the use of MAP-fabrication machine,

I. A two-step casting process will be used to fabricate Ca2+/Alg-sugar composite microneedles. Firstly, the microneedle matrix loaded with therapeutic protein or drug is poured into a refrigerated centrifuge at 10,000 rpm and 20 °C for 10 min to fill the porous container of the microneedle mould and form a thicker pre-casting microneedle baseplate.
II. Then, the preformed drug (lignocaine)-loaded microneedle preforming plate will be loaded into the machine and go through stamping, forming hardening to become a final microneedle array patch as shown in a schematic diagram in Figure 3

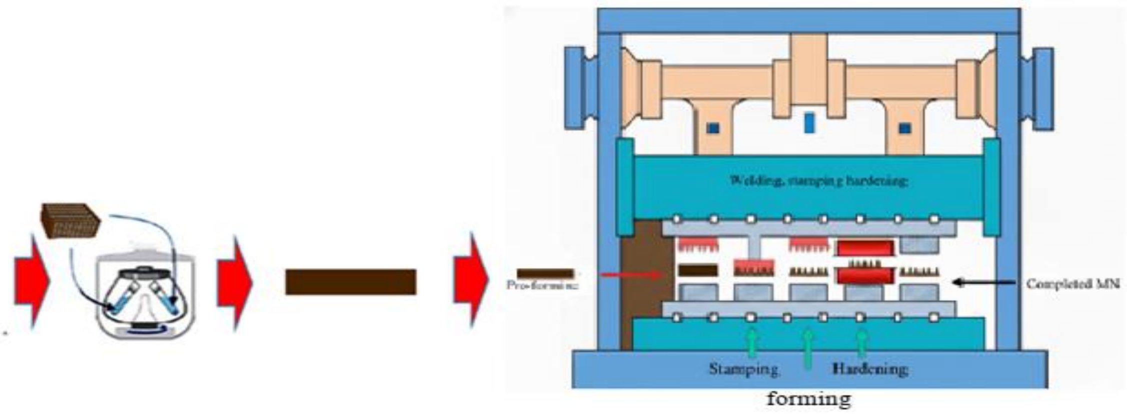

Sugar compounds such as sucrose, trehalose, and maltose will be used to mixed with the dedicated drug/s and the mixture will go through a centrifuge process in a perforated container to form a thicker pre-casting microneedle baseplate

Preformed drug loaded microneedle preforming plate will be loaded to the machine and go through stamping, forming hardening to become a final microneedle array patch

Figure 3: The microneedle matrix composite will have filled into a porous container to form a pre-casted microneedle plate, the plate will be used to form microneedle tips and baseplate after a series of stamping, forming, hardening processes. The microneedle patch is made in a prototype lab in Alnair Incorporated, Tokyo, Japan, which is the collaborator in this study. The microneedles were individually heat treated at 120°C for sanitation before packaging (Authors' own image).

4.12 Trial Conduct

4.12.1 Pharmacokinetic (PK) Study Conducts

Therefore, for this project, we will conduct a subproject as a Phase 1 trial to assess the safety and tolerability of 12.5mg lignocaine-embedded microneedle on a small number of adult patients (20 patients; 10 males and 10 females) without hepatic or renal dysfunction. All participants in this Phase 1 trial will be cataract patients recruited from the HCTM ophthalmology outpatient clinic. Pre-treatment fasting is not required for all participants.

On the day of the study, each potential participant will be screened for study eligibility based on our pre-specified inclusion and exclusion criteria. An interim abridged medical history will be taken from each participant and their list of medications will be reviewed. Vital signs (systolic and diastolic blood pressures, oral temperature, pulse and respiratory rates) will be taken and targeted clinical examinations will be performed by the medical officers to assess the overall health of the participants.

First, an intravenous cannula will be placed at the dorsum of the hand, and routine blood samples will be taken for investigation. Additionally, approximately 3.0 mL of venous blood samples will then be withdrawn at t = 0. Then the 12.5mg lignocaine-impregnated microneedles will be applied to the antecubital fossa, after which blood will be drawn from the earlier inserted intravenous cannula at t = 30, 60, 90, 120, 180 minutes and collected into separate 3.5-ml plastic blood collection tubes with accelerator & separator gel (BD™, New Jersey, USA). Heparinised saline will be periodically infused to ensure that the cannula lumen remains patent throughout the sampling periods. The blood samples will then be sent to *Jabatan Kimia Malaysia* (Malaysian Chemistry Department) for lignocaine concentration measurements using the validated methodology of Gas Chromatography Nitrogen Phosphorus Detector (GC-NPD).

In comparison with the 12.5mg lignocaine-embedded microneedle, a similar approach to the one above will be used to test the systemic absorption of EMLA in a group of 20 additional patients. This will utilise the method employed in the previous paediatric trial, i.e., 5% EMLA cream 1 FTU enhanced by a microneedle patch.

a) Determining Serum Lignocaine Concentration using Gas Chromatography-Nitrogen Phosphorus Detector (GC-NPD): A Brief Protocol

One (1) mL of the blood will be taken from the collection tube and alkalinised with NaOH solution of pH 12. The internal standard, Methaqualone and the organic solvent, Chlorobutane, will then be added to the mixture. The mixture will be subsequently mixed using a roller mixer and then centrifuged to extract the organic layer, which will be subsequently concentrated from the partition. A clean-up solution, hexane-ethanol, will then be added to the sample mixture, which will be vortexed and centrifuged again. The bottom organic layer will then be moved into another tube and it will be evaporated to complete dryness under nitrogen gas flow at room temperature. The residue will be subsequently reconstituted using absolute ethanol prior to loading it into the GC-NPD system.

Lignocaine in the blood matrix will be spiked based on the level below or within the range of the therapeutic level. Besides, the response of the drug in gas chromatography (GC) (i.e. the resolution and the peak in GC) will be taken into consideration. In normal practice, lignocaine at the amount of 0.5 parts per million (ppm) will be spiked and a lower amount of lignocaine (0.3 ppm) will be used for quality control, which are based on previous recommendations by Winek et al (Lignocaine: Therapeutic: 1.5-5.0 ppm; Toxic: 7-20 ppm; Lethal:>25 ppm).[70] For calibration, a 1-point calibration to estimate serum lignocaine concentration will be used. A series of 1-point calibrations will also be carried out whenever serum lignocaine concentration exceeds the therapeutic range.

b) Post-intervention Monitoring and Pharmacokinetics Data Analysis

The participants will be allowed to return home after the last blood sample is taken at t=180. The participants will be further monitored for any adverse events (AEs) such as redness, pain, itchiness, blistering, etc (local reactions) and light-headedness, euphoria, tinnitus, diplopia etc. (systemic reactions), serious adverse events (SAEs) and suspected unexpected serious adverse reactions (SUSARs) for up to 48 hours via telephone calls.

The pharmacokinetic data will first be summarised in mean/standard deviation or median/interquartile range for continuous data, and in counts and percentages for categorical data. The pharmacokinetic parameters (AUC_{inf}, AUC_t, C_{max}, C_{min}, t_{max}, $t_{1/2}$, volume of distribution (V_d), Clearance (Cl)) of lignocaine will be evaluated using blood samples obtained at times t=0, 30, 60, 90, 120, and 180 minutes after the application of lignocaine-impregnated MN patch. The intraindividual and interindividual variations of the pharmacokinetic parameters will be evaluated using the coefficient of variation (CV), and these will be classified as low (CV≤10%), moderate (CV ≈ 25%) and high (CV>40%).[71] The pharmacokinetic data will be analysed using the non-linear mixed effect models based on the two-compartmental model which will be implemented on NONMEM® version VI (Icon Development Solutions, Ellicott City, Maryland, USA). The influence of clinically relevant covariates such as participant's age, gender, BMI and others on pharmacokinetic parameters will be evaluated in a stepwise fashion. First-order conditional likelihood (FOCE INTER on NONMEM) will be used to fit the data, and model selection will be dependent upon the likelihood ratio test, the estimates of pharmacokinetic parameters and their 95% confidence intervals and goodness-of-fit measures.

4.12.2 Pharmacodynamic (PD) Study Conduct

a) Randomisation Procedure and Blinding (Masking) of Trial Participants

For random allocation, a block randomisation procedure with varying block sizes (permuted block) will be utilised to guarantee that both intervention groups will have an equal number of trial participants. This will be carried out by the trial statistician using the R package, blockrand version 1.50, which will be implemented on the R platform.[72] The list of generated random numbers will be used to allocate the study participants to either the intervention or the control arm. The allocation sequence generated will be stored in a password-protected document, accessible only to the statistician, to maintain allocation concealment. To further ensure the

adequacy of allocation concealment, the randomisation code will not be revealed until the potential trial participants have been definitively enrolled into the trial, which will be after all baseline measurements are made and all eligibility criteria are deemed fulfilled by the study recruiters. In addition, allocation concealment is further safeguarded by ensuring the identity of the allotted treatment is only revealed to the interventionist (i.e. the person who will be administering the intervention) via secure telephone calls (central randomisation). Consecutive recruitments will be made until the final intended sample size is achieved.

For this study, the outcome assessors and care providers (who may be the same individual) will be masked to the identity of interventions (single blinding/single masking). Only the statistician and interventionist/procedurist will be unmasked to the study interventions. Furthermore, a unique ID code will be generated to indicate each treatment sequence assignment and utilised to ensure that the unintentional/intentional unmasking of one trial participant does not compromise the integrity of blinding for the rest of the study participants. The primary unblinded trial personnel (subjects, the statistician and the procedurist/interventionist) are instructed not to divulge the identity of the allotted treatments to other blinded trial personnel. The success of blinding will be determined by asking the blinded trial participants to guess the identity of the interventions received and then comparing the results obtained with what would be anticipated by chance. Blinding indices such as James' Blinding Index or Bang's Blinding Index could also be calculated to objectively assess whether blinding has been successfully achieved in this trial.[73,74]

b) Administration of Lignocaine-embedded Microneedle (intervention) and EMLA (control) patches

Prior to the administration of intervention/control, relevant clinic-demographic profiles (age, gender, ethnicity, anthropometric measurements, presence of comorbidities,) will be recorded and entered in the case report forms (CRFs) that are specifically designed for this study. This research study utilises lignocaine-embedded microneedles. The comparison of pharmacodynamic properties (i.e. efficacy) between 12.5 mg lignocaine delivered through direct embedment within the microneedle matrix and standard 5% EMLA dermal patch containing 1 finger-tip-unit (1 FTU = 0.5g) of 12.5mg lignocaine and 12.5mg prilocaine will be assessed via VAS score and skin algesimeter index for the pain induced by venepuncture.

The window period for lignocaine to be effective will be based on usual clinical practice observations, where it is typically applied 30 minutes prior to venepuncture. The rationale behind it is due to logistical issues and for the daycare's operational convenience. Nevertheless, in a busy clinical setting, the application time is sometimes shortened to 15 minutes for a slight anaesthetic effect. Thus, we postulate that, with the aid of microneedle, the time to onset of action for lignocaine could be greatly reduced, resulting in a much-reduced pain sensation when the clinical assessment is carried out 30 minutes after treatment application.

The administrator of interventions (procedurist) will identify and draw a 1cm × 1cm grid at the antecubital fossa, which will serve as an ideal site for cannulation. The procedurist will then apply the lignocaine-impregnated microneedle patch. After a 30-minute application time, the attending medical officer will perform venepuncture using a 21-gauge (G) hypodermic needle inserted into the vein beneath the analgesic patch.

For the participants allotted to the standard 5% EMLA dermal patch, 1 FTU of 5% EMLA cream will be applied and covered with a piece of adhesive to form a dermal patch. This will be applied for 30 minutes similarly on the antecubital fossa. This application delivers an equivalent of 12.5mg lignocaine and 12.5mg prilocaine.

During the trial day, the participants will not be allowed to take any analgesic medications (NSAID, Opioids, Paracetamol) since they will modulate the level of pain experienced by the participants due to the received interventions. Other medications and concomitant care will be permitted during the trial.

c) Pain Assessment

The study participants will first be guided on the operating manual for a 10-points, 100mm VAS pain score by an outcome assessor. The participants will be presented with a ruler that contains 100-mm slots with "No Pain" written on the left side and "Worst Pain" on the opposite right side. The study participants will then be asked to move and place the slider in the slot that accurately describes his/her pain at the following time points: 1) within 5 minutes after application of lignocaine-impregnated MN patch and before venepuncture/IV cannulation (baseline VAS score); 2) within 5 minutes after venepuncture/IV cannulation. The investigator/outcome assessor, who is blinded to the subject intervention arm, will record the location of the slot where the slider is placed in millimetres (mm), clearly printed on the ruler's reverse side, and this will be the participant's VAS score. Throughout the process, there will be a trained investigator will be available to assist with the verification of the pain scale and to aid the participants who require additional assistance.

For a subset of randomly selected patients, before applying the MN patch and EMLA Cream, the patients will be attached with the PainMonitor™ (Med-Storm Innovation AS, Oslo, Norway) device, whereby the electrodes will be attached to the hypothenar eminence of the opposite hand not receiving the vein cannulation. The procedurist will set up this machine and application before the interventions are commenced. The skin conductance peaks (in microSiemens (μS) and the skin algesimeter index (in microSiemens per second (μS/s)) will be recorded by the outcome assessor, who will be blinded to the subject intervention arm. Those parameters indicate the skin's sympathetic nerve block induced by the topical anaesthetic. The measurement time points start from the point of intervention and the recordings continued for at least 15 seconds.

4.12.3 Clinical Data Collection

All study personnel (i.e. interventionist/procedurist, investigator/outcome assessors, healthcare providers) will receive training (e.g. use of open-ended questions when assessing VAS pain score and adverse events related to interventions) in VAS measurement, administration of lignocaine-embedded MN patch prior to trial commencement to standardise data collection, enhance data quality and reduce data inconsistency and measurement variability.

All data collected will be checked for data quality using double data entry practice and checking for sensible data range and format (e.g. integer for the number of adverse events experienced

within 24 hours of intervention). All paper-based CRFs will be stored in locked cabinets that are only accessible to the principal investigators, data manager and statistician and these will be maintained for 5 years after the trial ends. For quality control, periodic random check on a subset of CRFs will be carried out.

All data recorded on paper-based CRFs, which will then be transcribed into an SPSS spreadsheet in an .sav extension at the central site, conducted by statistician-trained data entry personnel. This will then be converted into a Stata-friendly file format (.dta extension) to aid statistical analysis. The dataset will be password-protected and is only accessible to the principal study researchers and statistician to prevent any intended or unintended breach of patient confidentiality. Backup datasets will be stored on password-protected thumb drives and cloud storage (GoogleDrive) that are again only accessible to the principal study investigators and statistician. The password for all datasets will be regularly changed to ensure maximum protection against any data breach.

4.12.4 Interventional Safety Assessment

We define adverse events (AE) as "an abnormal sign, symptom, laboratory test, syndromic combination of such abnormalities, untoward or unplanned occurrence (e.g. accident), or any unexpected deterioration of concurrent illness".[75] For serious AE (SAE), this is defined as "adverse events that result in the following outcomes: 1) death; 2) life-threatening AEs; 3) inpatient hospitalisation or prolongation of existing hospitalisation; 4) a persistence of significant incapacity or substantial disruption of the ability to conduct normal life functions, or a congenital anomaly or birth defect.[76]

We classify the likelihood of AEs / SAEs (unrelated, possible, probable, definite) based on Naranjo et al. classification.[77] All AEs/SAEs will be recorded and graded based on the Common Terminology Criteria for Adverse Events (CTCAE) Version 5 and the US FDA's Toxicity Grading Scale for Healthy Adults and Adolescents Volunteers Enrolled in Preventive Vaccine Clinical Trials. All AEs or SAEs can be classified into local skin reaction (pain, erythema, ecchymosis, swelling, itchiness, tenderness) or systemic reaction (fever, irritability, tiredness, anorexia, vomiting, tachycardia, seizure, hypotension).

All AEs will be recorded on the CRFs. The detailed characteristics, the time and dates of onset and disappearance, and the severity of AEs will be included in the CRFs. The study investigators will assess each participant experiencing AEs and they will receive appropriate treatments accordingly. The relationships between AEs and lignocaine-embedded microneedles will be evaluated by the investigators and classified as either unrelated, possible, probable or definite based on Naranjo et al. classification. AEs are considered unexpected AEs when the AEs are not previously observed and not reported in the Investigator’s Brochure or standard lignocaine package insert. Any incidence of AEs or SAEs classified as possibly, probably and definitely linked to lignocaine-embedded microneedles will be monitored until the resolution of AEs/SAEs is complete or the Investigator deems that the AEs or SAEs have become stable or irrevocable.

All AEs of grade 3 and above will be reported to the JEPUKM within 5 business days. All SAEs (including Serious Unexpected Suspected Adverse Events (SUSARs)) will be reported within 24 hours of occurrence (expedited reporting) to the JEPUKM. If AEs / SAEs occur or

are still ongoing by the end of the study period, the study participants will still be continuously followed up until complete resolution of AEs / SAEs which will take the following form: 1) additional participant visit to the trial centre / hospital; 2) telephone calls to the subjects; 3) additional reporting in the form of letters from the treating physicians.

Participant enrolment, intervention allocation and administration will be stopped if one of the following occurs (study halting criteria):

a) Death related to lignocaine-impregnated MN patch

b) Any participant experiences bronchospasm, laryngospasms or anaphylaxis within 24 hours post lignocaine-impregnated MN patch

c) Any SAE related to lignocaine-impregnated MN patch

d) Any AE of grade 3 and above or any SAE that cannot obviously be attributed to other causes

e) Any study participant who develops an abscess, ulceration or erosion at the site(s) of the lignocaine-impregnated MN patch

To ensure the independence of safety monitoring, all recorded safety data will be reviewed by JEPUKM, which functions as an independent Data Safety Monitoring Board (DSMB) for our trial.

4.13 Operational Definitions of Study Variables

a) Independent variables

i) Age: The age of a study participant at the first study visit. The variable will be measured in years and month and modelled as a continuous numerical variable and will not be categorised into separate age groups.

ii) Gender: A categorical variable that will be recorded in the SPSS data frame as 0 = female (base category) and 1 = male. Missing data shall be recorded as 999

iii) Ethnicity: A categorical variable that will be recorded in the SPSS data frame as 0 = Malay (base category); 1 = Chinese; 2 = Indian; and 3 = Other ethnicity. Missing data shall be recorded as 999.

iv) Body mass index (BMI): A continuous numerical variable that is calculated using the standard body mass index formula; BMI = kg / m^2. This variable will be categorised according to a widely used BMI classification[78, 79]:

<18.5= underweight (SPSS code: -1)
18.5-24.9= normal BMI (SPSS code: 0; base category)
25.0-29.9= Overweight (SPSS code: +1)
≥30 = Obese (SPSS code: +2)

The categorised variable will then be used as a predictor variable for statistical modelling

purposes. Missing data will be recorded as 999 in the SPSS spreadsheet.

v) Intervention groups: A categorical variable representing the types of interventions received by the study participants in each study visit. This variable will be recorded in the SPSS data frame as 0 = 5% EMLA dermal patch only group (control, base category, 30 minutes); 1 = Lignocaine-Embedded Microneedle Patch (30 minutes)

vi) Baseline VAS score: A continuous numerical variable that will be measured before the administration of intervention. This will be used as a predictor variable to control the confounding effect of heterogenous baseline VAS scores among study participants. Missing data will be recorded as 999 in the SPSS spreadsheet.

vii) Baseline pain score obtained via PainMonitor™ device: A continuous numerical variable that will be measured before the administration of the intervention. This will be used as a predictor variable to control the confounding effect of heterogeneous baseline VAS scores among study participants. Missing data will be recorded as 999 in the SPSS spreadsheet.

b) Dependent (outcome variables)

i) VAS score (30 minutes post-intervention application): A continuous numerical variable that will be measured during each visit; 30 minutes after lignocaine-embedded microneedle or 5% EMLA application. Missing data will be recorded as 999 in the SPSS spreadsheet.

ii) Pain score from PainMonitor™ device (30-minute post intervention application): A continuous numerical variable that will be measured during each visit: 30 minutes after either lignocaine-embedded microneedle or 5% EMLA application. Missing data will be recorded as 999 in the SPPS spreadsheet.

4.14 Ethical Issues

Voluntary written informed consent will be obtained from each study participant. This study will be conducted in accordance with the principles of ethics in human research as stipulated by the Declaration of Helsinki (18th World Medical Association General Assembly, 1964), the Good Clinical Practice (GCP) guidelines, and ISO14155:2020 Clinical Investigation for Medical Devices for Human Subjects. Ethical approval will be obtained from the UKM Research Ethics Committee (Human) (JEPUKM).

All research participants will sign informed consent forms prior to their study participation. The participants will be made aware that their participation is completely voluntary and they can withdraw from the study at any time point. The research participants will also be notified that their decisions to withdraw from the study will not jeopardise their current or subsequent treatments and healthcare services received. To ensure the confidentiality of patient information, each participant will be assigned an anonymous research ID code that will be used for data storage and analysis. The data will solely be made available to the research team members and access to the storage may only be granted by the principal investigators.

To enhance the transparency of reporting, the trial will be registered at the Clinical Trials Registry (https://clinicaltrials.gov/) and the Malaysian National Medical Research Registry (NMRR) (https://nmrr.gov.my/). The full trial protocol will be made available in the same trial registries and prepared according to the Consolidated Standards of Reporting Trials (CONSORT) guideline for parallel design and the 2013 Standard Protocol Items: Recommendations for Interventional Trials (SPIRIT) statement. Any future protocol modifications will be submitted first to the JEPUKM for approval and the list of protocol changes will be made available to the public via both the Clinical Trials and NMRR registries.

The trial data with study participant identification numbers removed (Hospital RN, Identity Card (IC) numbers, subject's identification number (SIDNO) will be made available to the public via Harvard Dataverse repository for research data (https://dataverse.harvard.edu) to ensure the transparent dissemination of study findings and adherence to recommendations made by leading medical journal editors for the future publication of this research.

4.15 Statistical Analysis

Data analysis will be performed using Statistical Package for Social Science (SPSS™) (IBM Corp. Released 2020. IBM Statistics for Windows, Version 27.0, Armonk, NY: IB Corp) and STATA™ version 15 (StatCorp. 2017. Stata Statistical Software: Release 15. College Station, Texas: StataCorp LP). Our primary analysis will be based on the intention-to-treat (ITT) principle by which all trial participants will be analysed according to their original intended treatment assignment. For missing observations, we will use the multiple imputation method to fill in the missing data, assuming the missing at random (MAR) mechanism. To assess the robustness of the results, we will also conduct sensitivity analysis by comparing the results obtained using complete case analysis (i.e. including patients with complete observations) with full case (i.e. patients with complete and imputed observations for missing data) analysis.

The differences in terms of the outcome measures between the groups will be statistically assessed using an independent t-test if the parametric assumption is met (i.e. the data are normally distributed) or the Mann-Whitney test if the data are non-normally distributed. To control and adjust the effects of confounding variables such as baseline pain scores, body mass index (BMI), gender of the patients, multiple linear regression analysis will be used. Variable selection will be based on a mixture of strategies: 1) stepwise regression based on Akaike Information Criterion (AIC), Bayesian Information Criterion (BIC); 2) purposeful selection of covariates; 3) clinical domain knowledge. Effect modification will be assessed by creating and assessing the significance of the statistical interaction terms. The goodness of fit of the model will be evaluated using the coefficient of multiple determination, R2.

Model assumptions (linearity, independence, normality, and homoscedasticity of residuals) will be assessed using studentised residual vs predicted values scatter plots and Durbin Watson statistics. Box-Cox transformation with carefully selected exponent (lambda) will be employed in the presence of skewness in the outcome variables. The presence of influential observations will be suspected in the presence of large leverage (extreme value in the x space) and residual values (extreme value in the y space) for any outlying observations. This will be further confirmed using influential diagnostic measures such as Cook's distance[80], dfFITS and dfBeta.[81] The significance threshold will be set at 0.05 and 95% confidence intervals will be presented for each effect estimate.

TRIAL WORKFLOW

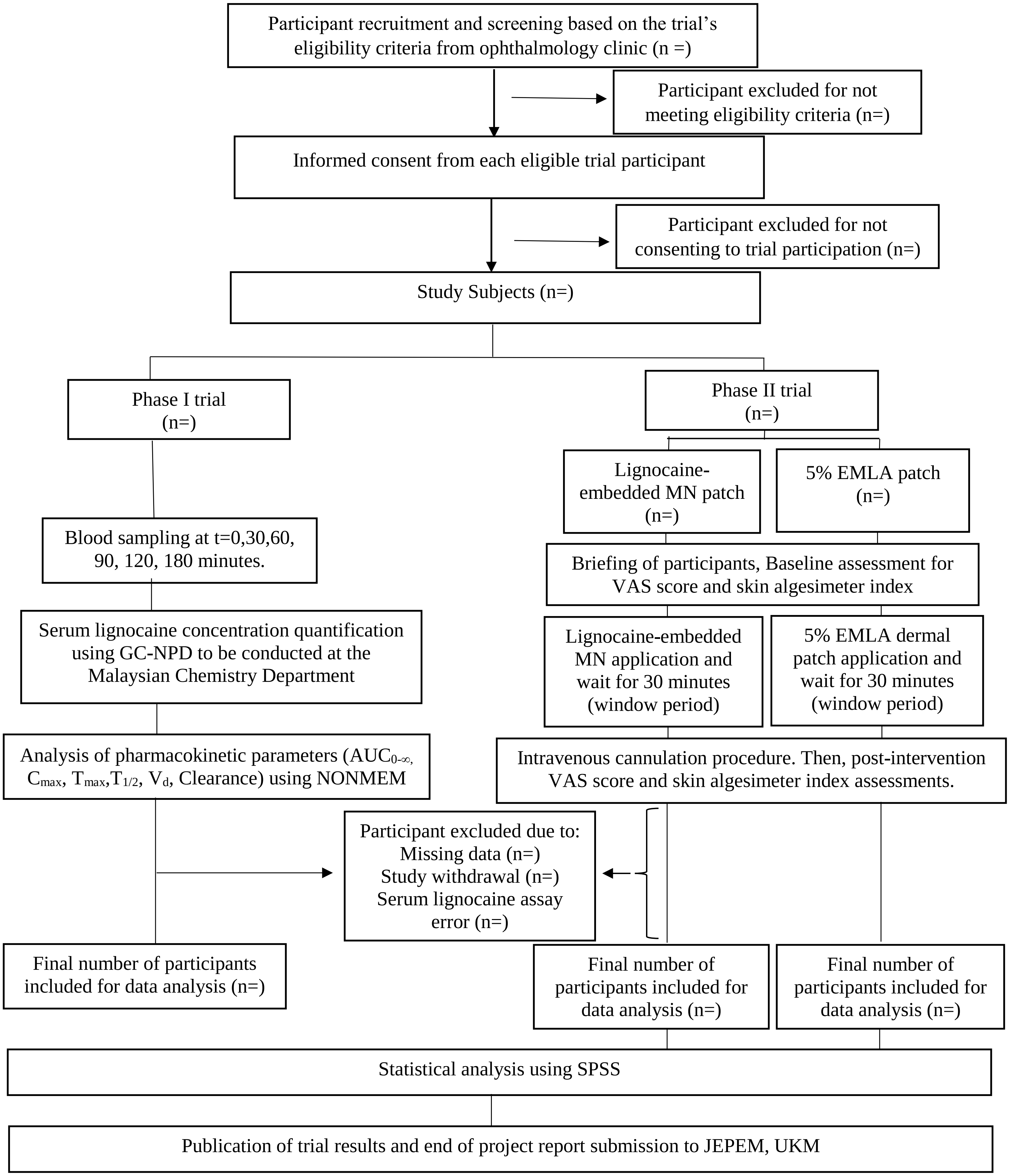

Figure 1: Anticipated trial workflow based on the 2010 CONSORT statement flowchart

GANTT CHART

**Project activities**	**Timeline**																								
	2022		2023												2024										
	11	12	1	2	3	4	5	6	7	8	9	10	11	12	1	2	3	4	5	6	7	8	9	10	11
Research application for ethics approval																									
Trial subject recruitment																									
Serum assays for lignocaine levels																									
Data analyses and interpretation																									
Data presentation, Manuscript preparation, Final project report, and Publication																									
End of project																									

BUDGET

No.	Description	Unit price (RM)		Quantity	Price (RM)	
1	PROFESSIONAL SERVICE					
1.1	Lignocaine quantitation assay with GC-NPD	125	sample	120	15000	00
	Total				15000	

REFERENCES

1. Alkilani AZ, McCrudden MT, Donnelly RF. Transdermal Drug Delivery: Innovative Pharmaceutical Developments Based on Disruption of the Barrier Properties of the stratum corneum. Pharmaceutics. 2015;7(4):438-70. doi: 10.3390/pharmaceutics7040 438.

2. Jeong WY, Kwon M, Choi HE, Kim KS. Recent advances in transdermal drug delivery systems: a review. Biomater Res. 2021;25(1):24. doi: 10.1186/s40824-021-00226-6.

3. Lee BM, Lee C, Lahiji SF, Jung UW, Chung G, Jung H. Dissolving Microneedles for Rapid and Painless Local Anesthesia. Pharmaceutics. 2020;12(4):366. doi: 10.3390/pharmaceutics12040366.

4. Murthy SN, Sammeta SM, Bowers C. Magnetophoresis for enhancing transdermal drug delivery: Mechanistic studies and patch design. J Control Release. 2010;148(2):197-203. doi: 10.1016/j.jconrel.2010.08.015.

5. Zempsky WT, Sullivan J, Paulson DM, Hoath SB. Evaluation of a low-dose lidocaine iontophoresis system for topical anesthesia in adults and children: a randomized, controlled trial. Clin Ther. 2004;26(7):1110-9. doi: 10.1016/s0149-2918(04)90183-x.

6. Kim TY, Jung DI, Kim YI, Yang JH, Shin SC. Anesthetic effects of lidocaine hydrochloride gel using low frequency ultrasound of 0.5 MHz. J Pharm Pharm Sci. 2007;10(1):1-8.

7. Benson HAE, Grice JE, Mohammed Y, Namjoshi S, Roberts MS. Topical and Transdermal Drug Delivery: From Simple Potions to Smart Technologies. Curr Drug Deliv. 2019;16(5):444-460. doi: 10.2174/1567201816666190201143457.

8. Yousef H, Alhajj M, Sharma S. Anatomy, Skin (Integument), Epidermis. [Updated 2021 Nov 19]. In: StatPearls [Internet]. Treasure Island (FL): StatPearls Publishing; 2022 Jan-. Available from: https://www.ncbi.nlm.nih.gov/books/NBK470464/

9. Ali S, Shabbir M, Shahid N. The structure of skin and transdermal drug delivery system - A review. Research Journal of Pharmacy and Technology. 2015; 8(2):103-109. doi: 0.5958/0974-360X.2015.00019.0

10. Duarah S, Sharma M, Wen J. Recent advances in microneedle-based drug delivery: Special emphasis on its use in paediatric population. Eur J Pharm Biopharm. 2019;136:48-69. doi: 10.1016/j.ejpb.2019.01.005.

11. Liu X, Kruger P, Maibach H, Colditz PB, Roberts MS. Using skin for drug delivery and diagnosis in the critically ill. Adv Drug Deliv Rev. 2014 ;77:40-9. doi: 10.1016/j.addr.2014.10.004.

12. Brown MB, Martin GP, Jones SA, Akomeah FK. Dermal and transdermal drug delivery systems: current and future prospects. Drug Deliv. 2006;13(3):175-87. doi: 10.1080/10717540500455975.

13. Andrews SN, Jeong E, Prausnitz MR. Transdermal delivery of molecules is limited by full epidermis, not just stratum corneum. Pharm Res. 2013;30(4):1099-109. doi: 10.1007/s11095-012-0946-7.

14. Ita K. Transdermal Delivery of Drugs with Microneedles-Potential and Challenges. Pharmaceutics. 2015;7(3):90-105. doi: 10.3390/pharmaceutics7030090.

15. Prausnitz MR, Langer R. Transdermal drug delivery. Nature biotechnology. 2008;26(11): 1261-1268

16. Waghule T, Singhvi G, Dubey SK, Pandey MM, Gupta G, Singh M, Dua K. Microneedles: A smart approach and increasing potential for transdermal drug delivery system. Biomed Pharmacother. 2019;109:1249-1258. doi: 10.1016/j.biopha.2018.10.078.

17. Li J, Zeng M, Shan H, Tong C. Microneedle Patches as Drug and Vaccine Delivery Platform. Curr Med Chem. 2017;24(22):2413-2422. doi: 10.2174/0929867324666170526124053.

18. Xu J, Xu D, Xuan X, He H. Advances of Microneedles in Biomedical Applications. Molecules. 2021;26(19):5912. doi: 10.3390/molecules26195912.

19. Zhang Y, Jiang G, Yu W, Liu D, Xu B. Microneedles fabricated from alginate and maltose for transdermal delivery of insulin on diabetic rats. Mater Sci Eng C Mater Biol Appl. 2018;85:18-26. doi: 10.1016/j.msec.2017.12.006.

20. Larrañeta E, Lutton REM, Woolfson AD, Donnelly RF. Microneedle arrays as transdermal and intradermal drug delivery systems: Materials science, manufacture and commercial development. Materials Science and Engineering R: Reports. 2016;104:1-32. https://doi.org/10.1016/j.mser.2016.03.001.

21. Kolli CS, Banga AK. Characterization of solid maltose microneedles and their use for transdermal delivery. Pharm Res. 2008;25(1):104-13. doi: 10.1007/s11095-007-9350-0.

22. Sartawi Z, Blackshields C, Faisal W. Dissolving microneedles: Applications and growing therapeutic potential. J Control Release. 2022;348:186-205. doi: 10.1016/j.jconrel.2022.05.045.

23. Rzhevskiy A, Popov A, Pavlov C, Anissimov Y, Zvyagin A, Levin Y, Kochba E. Intradermal injection of lidocaine with a microneedle device to provide rapid local anaesthesia for peripheral intravenous cannulation: A randomised open-label placebo-controlled clinical trial. PLoS One. 2022;17(1):e0261641. doi: 10.1371/journal.pone.0261641.

24. Ornelas J, Foolad N, Shi V, Burney W, Sivamani RK. Effect of Microneedle Pretreatment on Topical Anesthesia: A Randomized Clinical Trial. JAMA Dermatol. 2016;152(4):476-7. doi: 10.1001/jamadermatol.2015.5544.

25. Gupta J, Denson DD, Felner EI, Prausnitz MR. Rapid local anesthesia in humans using minimally invasive microneedles. Clin J Pain. 2012;28(2):129-35. doi: 10.1097/AJP.0b013e318225dbe9.

26. Lidocaine Hydrochloride and Epinephrine Injection USP [Package Insert]. Quebec, Canada: Pfizer Canada ULC; 2006.

27. Olschewski A, Hempelmann G, Vogel W, Safronov BV. Blockade of Na+ and K+ currents by local anesthetics in the dorsal horn neurons of the spinal cord. Anesthesiology. 1998;88(1):172-9. doi: 10.1097/00000542-199801000-00025.

28. Edwards DJ, Lalka D, Cerra F, Slaughter RL. Alpha1-acid glycoprotein concentration and protein binding in trauma. Clin Pharmacol Ther. 1982;31(1):62-7. doi: 10.1038/clpt.1982.10.

29. Tesseromatis C, Kotsiou A, Tsagataki M, Tigka E, Vovou J, Alevizou A, Perisanidis C, Saranteas T, Karakitsos D, Karabinis A, Kostopanagiotou G. In vitro binding of lidocaine to liver tissue under the influence of propranolol: another mechanism of interaction? Eur J Drug Metab Pharmacokinet. 2007;32(4):213-7. doi: 10.1007/BF03191006.

30. Routledge PA, Stargel WW, Wagner GS, Shand DG. Increased alpha-1-acid glycoprotein and lidocaine disposition in myocardial infarction. Ann Intern Med. 1980;93(5):701-4. doi: 10.7326/0003-4819-93-5-701.

31. Barry M, Keeling PW, Weir D, Feely J. Severity of cirrhosis and the relationship of alpha 1-acid glycoprotein concentration to plasma protein binding of lidocaine. Clin Pharmacol Ther. 1990;47(3):366-70. doi: 10.1038/clpt.1990.41.

32. McNamara PJ, Slaughter RL, Visco JP, Elwood CM, Siegel JH, Lalka D. Effect of smoking on binding of lidocaine to human serum proteins. J Pharm Sci. 1980;69(6):749-51. doi: 10.1002/jps.2600690646.

33. Lerman J, Strong HA, LeDez KM, Swartz J, Rieder MJ, Burrows FA. Effects of age on the serum concentration of alpha 1-acid glycoprotein and the binding of lidocaine in pediatric patients. Clin Pharmacol Ther. 1989;46(2):219-25. doi: 10.1038/clpt.1989.129.

34. Routledge PA, Shand DG, Barchowsky A, Wagner G, Stargel WW. Relationship between alpha 1-acid glycoprotein and lidocaine disposition in myocardial infarction. Clin Pharmacol Ther. 1981;30(2):154-7. doi: 10.1038/clpt.1981.141.

35. Burm AG, de Boer AG, van Kleef JW, Vermeulen NP, de Leede LG, Spierdijk J, Breimer DD. Pharmacokinetics of lidocaine and bupivacaine and stable isotope labelled analogues: a study in healthy volunteers. Biopharm Drug Dispos. 1988;9(1):85-95. doi: 10.1002/bod.2510090109.

36. Thomson PD, Melmon KL, Richardson JA, Cohn K, Steinbrunn W, Cudihee R, Rowland M. Lidocaine pharmacokinetics in advanced heart failure, liver disease, and renal failure in humans. Ann Intern Med. 1973;78(4):499-508. doi: 10.7326/0003-4819-78-4-499.

37. Huet PM, Lelorier J, Pomier G, Marleau D. Bioavailability of lidocaine in normal volunteers and cirrhotic patients. Clin Pharmacol Ther. 1979;25(2):229-30.

38. Bauer LA, Horn JR, Maxon MS, Easterling TR, Shen DD, Strandness DE Jr. Effect of metoprolol and verapamil administered separately and concurrently after single doses on liver blood flow and drug disposition. J Clin Pharmacol. 2000;40(5):533-43. doi: 10.1177/00912700022009152.

39. Prescott LF, Adjepon-Yamoah KK, Talbot RG. Impaired Lignocaine metabolism in patients with myocardial infarction and cardiac failure. Br Med J. 1976;1(6015):939-41. doi: 10.1136/bmj.1.6015.939.

40. Wang JS, Backman JT, Taavitsainen P, Neuvonen PJ, Kivistö KT. Involvement of CYP1A2 and CYP3A4 in lidocaine N-deethylation and 3-hydroxylation in humans. Drug Metab Dispos. 2000;28(8):959-65.

41. Huang W, Lin YS, McConn DJ 2nd, Calamia JC, Totah RA, Isoherranen N, Glodowski M, Thummel KE. Evidence of significant contribution from CYP3A5 to hepatic drug metabolism. Drug Metab Dispos. 2004;32(12):1434-45. doi: 10.1124/dmd.104.001313.

42. Ha HR, Candinas R, Stieger B, Meyer UA, Follath F. Interaction between amiodarone and lidocaine. J Cardiovasc Pharmacol. 1996;28(4):533-9. doi: 10.1097/00005344-199610000-00009.

43. Isohanni MH, Neuvonen PJ, Olkkola KT. Effect of fluvoxamine and erythromycin on the pharmacokinetics of oral lidocaine. Basic Clin Pharmacol Toxicol. 2006;99(2):168-72. doi: 10.1111/j.1742-7843.2006.pto_482.x.

44. Olkkola KT, Isohanni MH, Hamunen K, Neuvonen PJ. The effect of erythromycin and fluvoxamine on the pharmacokinetics of intravenous lidocaine. Anesth Analg. 2005;100(5):1352-1356. doi: 10.1213/01.ANE.0000148123.79437.F9

45. Orlando R, Piccoli P, De Martin S, Padrini R, Floreani M, Palatini P. Cytochrome P450 1A2 is a major determinant of lidocaine metabolism in vivo: effects of liver function. Clin Pharmacol Ther. 2004;75(1):80-8. doi: 10.1016/j.clpt.2003.09.007.

46. Narang PK, Crouthamel WG, Carliner NH, Fisher ML. Lidocaine and its active metabolites. Clin Pharmacol Ther. 1978;24(6):654-62. doi: 10.1002/cpt1978246654.

47. Thomson AH, Elliott HL, Kelman AW, Meredith PA, Whiting B. The pharmacokinetics and pharmacodynamics of lignocaine and MEGX in healthy subjects. J Pharmacokinet Biopharm. 1987;15(2):101-15. doi: 10.1007/BF01062338.

48. Strong JM, Mayfield DE, Atkinson AJ Jr, Burris BC, Raymon F, Webster LT Jr. Pharmacological activity, metabolism, and pharmacokinetics of glycinexylidide. Clin Pharmacol Ther. 1975;17(2):184-94. doi: 10.1002/cpt1975172184.

49. Wójcicki J, Kozłowski K, Droździk M, Wójcicki M. Lidocaine elimination in patients with liver cirrhosis. Acta Pol Pharm. 2002;59(4):321-4.

50. Rademaker AW, Kellen J, Tam YK, Wyse DG. Character of adverse effects of prophylactic lidocaine in the coronary care unit. Clin Pharmacol Ther. 1986;40(1):71-80. doi: 10.1038/clpt.1986.141.

51. Weinberg L, Peake B, Tan C, Nikfarjam M. Pharmacokinetics and Pharmacodynamics of Lignocaine: A Review. World Journal of Anesthesiology. 2015;4(2):17-29. doi: 0.5313/wja.v4.i2.17.

52. Kirkland DJ, Sheil ML, Streicker MA, Johnson GE. A weight of evidence assessment of the genotoxicity of 2,6-xylidine based on existing and new data, with relevance to safety of lidocaine exposure. Regul Toxicol Pharmacol. 2021;119:104838. doi: 10.1016/j.yrtph.2020.104838.

53. Davies PS, Galer BS. Review of lidocaine patch 5% studies in the treatment of postherpetic neuralgia. Drugs. 2004;64(9):937-47. doi: 10.2165/00003495-200464090-00002.

54. Gimbel J, Linn R, Hale M, Nicholson B. Lidocaine patch treatment in patients with low back pain: results of an open-label, nonrandomized pilot study. American Journal of Therapeutics. 2005;12(4):311-9. doi: 10.1097/01.mjt.0000164828.57392.ba.

55. Sun Y, Li T, Wang N, Yun Y, Gan TJ. Perioperative systemic lidocaine for postoperative analgesia and recovery after abdominal surgery: a meta-analysis of randomized controlled trials. Dis Colon Rectum. 2012;55(11):1183-94. doi: 10.1097/DCR.0b013e318259bcd8. Erratum in: Dis Colon Rectum. 2013;52(2):271.

56. Zink KA, Mayberry JC, Peck EG, Schreiber MA. Lidocaine patches reduce pain in trauma patients with rib fractures. Am Surg. 2011;77(4):438-42. doi: 10.1177/000313481107700419.

57. Kim CH, Yoon JU, Lee HJ, Shin SW, Yoon JY, Byeon GJ. Availability of a 5% lidocaine patch used prophylactically for venipuncture- or injection-related pain in children. J Anesth. 2012;26(4):552-5. doi: 10.1007/s00540-012-1360-3.

58. Bai Y, Miller T, Tan M, Law LS, Gan TJ. Lidocaine patch for acute pain management: a meta-analysis of prospective controlled trials. Curr Med Res Opin. 2015 Mar;31(3):575-81. doi: 10.1185/03007995.2014.973484.

59. Pensado A, McGrogan A, White KAJ, Bunge AL, Guy RH, Delgado-Charro MB. Assessment of dermal bioavailability: predicting the input function for topical glucocorticoids using stratum corneum sampling. Drug Deliv Transl Res. 2022 Apr;12(4):851-861. doi: 10.1007/s13346-021-01064-8.

60. Supe S, Takudage P. Methods for evaluating penetration of drug into the skin: A review. Skin Res Technol. 2021 May;27(3):299-308. doi: 10.1111/srt.12968.

61. Finnin B, Walters KA, Franz TJ. Chapter 5: In Vitro Skin Permeation Technology. In: Benson HAE, Watkinson AC. Transdermal and Topical Drug Deliver: Principles and Practice. Hoboken, NJ; Wiley: 2012. p.99.

62. Ronnander P, Simon L, Spilgies H, Koch A. Modelling the in-vitro dissolution and release of sumatriptan succinate from polyvinylpyrrolidone-based microneedles. Eur J Pharm Sci. 2018 Dec 1;125:54-63. doi: 10.1016/j.ejps.2018.09.010..

63. Ronnander P, Simon L, Koch A. Experimental and mathematical study of the transdermal delivery of sumatriptan succinate from polyvinylpyrrolidone-based microneedles. Eur J Pharm Biopharm. 2020 Jan;146:32-40. doi: 10.1016/j.ejpb.2019.11.007.

64. Ita, K. Microneedles. London, UK; Academic Press: 2022. p.163.

65. Yadav PR, Han T, Olatunji O, Pattanayek SK, Das DB. Mathematical Modelling, Simulation and Optimisation of Microneedles for Transdermal Drug Delivery: Trends and Progress. Pharmaceutics. 2020 Jul 22;12(8):693. doi: 10.3390/pharmaceutics12080693.

66. Bartley EJ, Fillingim RB. Sex differences in pain: a brief review of clinical and experimental findings. Br J Anaesth. 2013 Jul;111(1):52-8. doi: 10.1093/bja/aet127.

67. Ogungbenro K, Aarons L. How many subjects are necessary for population pharmacokinetic experiments? Confidence interval approach. Eur J Clin Pharmacol. 2008;64(7):705-13. doi: 10.1007/s00228-008-0493-7.

68. Julious SA. Sample size of 12 per group rule of thumb for a pilot study. Pharmaceutical Statistics: The Journal of Applied Statistics in the Pharmaceutical Industry. 2005;4(4):287-91.

69. Yamamoto LG, Boychuk RB. A blinded, randomized, paired, placebo-controlled trial of 20-minute EMLA cream to reduce the pain of peripheral i.v. cannulation in the ED. Am J Emerg Med. 1998 Nov;16(7):634-6. doi: 10.1016/s0735-6757(98)90163-2.

70. Winek CL, Wahba WW, Winek CL Jr, Balzer TW. Drug and chemical blood-level data 2001. Forensic Sci Int. 2001;122(2-3):107-23. doi: 10.1016/s0379-0738(01)00483-2.

71. Rowland M, Tozer TN. Clinical pharmacokinetics and pharmacodynamics: Concepts and Applications. 4th edition. Philadelphia: Lippincott Williams and Wilkins; 2011.

72. Greg Snow. blockrand: Randomization for Block Random Clinical Trials. R Package version 1.5. 2022. https://CRAN.R-project.org/package=blockrand

73. James KE, Bloch DA, Lee KK, Kraemer HC, Fuller RK. An index for assessing blindness in a multi-centre clinical trial: disulfiram for alcohol cessation-a VA cooperative study. Stat Med. 1996 Jul 15;15(13):1421-34.

74. Bang H, Ni L, Davis CE. Assessment of blinding in clinical trials. Control Clin Trials. 2004 Apr;25(2):143-56. doi: 10.1016/j.cct.2003.10.016. PMID: 15020033.

75. Aronson JK. Medication errors: what they are, how they happen, and how to avoid them. QJM. 2009;102(8):513-21. doi: 10.1093/qjmed/hcp052.

76. Code of Federal Regulation, Title 21, Section 312.32 (21CFR312.32). (2020). Accessible from: https://www.accessdata.fda.gov/scripts/cdrh/cfdocs/cfcfr/cfrsearch.cfm?fr=312.32. Date of access: 05 / 06 / 2022.

77. Naranjo CA, Busto U, Sellers EM, Sandor P, Ruiz I, Roberts EA, Janecek E, Domecq C, Greenblatt DJ. A method for estimating the probability of adverse drug reactions. Clin Pharmacol Ther. 1981;30(2):239-45. doi: 10.1038/clpt.1981.154.

78. Must A, Spadano J, Coakley EH, Field AE, Colditz G, Dietz WH. The disease burden associated with overweight and obesity. JAMA. 1999;282(16):1523-9. doi: 10.1001/jama.282.16.1523.

79. Obesity: preventing and managing the global epidemic. Report of a WHO consultation. World Health Organ Tech Rep Ser. 2000;894:i-xii, 1-253.

80. Cook RD. Detection of Influential Observation in Linear Regression. Technometrics. 1997;19(1):15–18. https://doi.org/10.2307/1268249

81. Belsley DA, Kuh E, Welsch RE. Regression diagnostics: Identifying influential data and sources of collinearity. New York: Wiley; 1980.

82. Muhammad Irfan Abdul Jalal , Kai Shen Ooi , Kai Cheong Foo et al. Transdermal Maltose-Based Microneedle Patch as Adjunct to Enhance Topical Anesthetic before Intravenous Cannulation of Pediatric Thalassemic Patients Receiving Blood Transfusion: A Randomized Controlled Trial Protocol J Clin Med 2022 Sep 8;11(18):5291. doi: 10.3390/jcm11185291.

APPENDIX A: PATIENT INFORMATION SHEET AND CONSENT FORM (MALAY VERSION)

BORANG MAKLUMAT DAN KEIZINAN PESAKIT

Tajuk kajian: **Penyerapan ubat lignocaine secara jarum mikro transdermal berbanding pelekat EMLA untuk kurangkan sakit sebelum prosedur tusukan vena ambil darah bagi pesakit dewasa yang menghadiri klinik.**

Pengenalan:
Anda dijemput untuk menyertai satu kajian penyelidikan klinikal. Sebelum menyertai kajian ini, adalah penting untuk anda membaca dan memahami secara menyeluruh dengan maklumat yang disediakan dalam kajian ini, dimana kajian ini akan dijelaskan secara lisan kepada anda dan anda akan diberi peluang untuk mengemukakan soalan. Setelah anda benar-benar berpuas hati bahawa anda telah memehami kajian ini, dan ingin menyertai atau terus mengambil bahagian dalam kajian ini, anda diwajibkan menandatangni borang persetujuan yang dikemukakan selepas ini. Anda akan diberikan salinan borang maklumat pesakit dan borang persetujuan untuk dibawa pulang bersama anda.

Tujuan kajian:
Prosedur tusukan vena sering menghasilkan kesakitan dan merupakan pengalaman yang traumatik yang sering dialami oleh pesakit yang menerima rawatan dengan tetapan penjagaan kesihatan klinikal. Namun, kesakitan tusuk vena mampu dikurangkan dengan menyapu ubat bius setempat. Sebagai contoh, krim lignocaine, adalah salah satu cara yang tidak invasif dan sesuai untuk pembiusan permukaan kulit badan. Krim lignocaine memerlukan jangka masa sekitar 30 minit untuk berkesan. Walaubagaimanapun, dalam persekitaran klinikal yang amat sibuk, tempoh masa yang diambil untuk kesan krim lignocaine berfungsi selalunya dikurangkan kepada 15 minit. Oleh itu, salah satu sistem penghantaran ubat transdermal (TDDS) telah diperkenalkan dengan jarum mikro yang berkesan untuk meningkatkan keberkesanan ubat bius topikal diserap dengan lebih cepat. Jarum mikro gubalan prototaip yang masih dikaji dan belum didaftar merupakan suatu peralatan yang mangandungi jarum kecil diperbuat daripada gula (maltosa) yang boleh diserap oleh kulit badan dan seterusnya mencapai objektif untuk penghantaran ubat anestetik. Oleh itu, dengan kajian penyelidikan semasa ini, kami berhasrat untuk menyiasat keselamatan dan toleransi tampalan jarum mikro yang diresapi dengan lignocaine bagi pesakit yang memerlukan prosedur tusukan vena atau kanulasi intravena yang kerap. Selain itu, kajian ini juga bertujuan untuk mendapatkan maklumat awal berkenaan keberkesanan jarum mikro yang diresapi lignocaine dalam mengurangkan kesakitan yang disebabkan oleh tusukan vene dan kanulasi intravena.

Bagaimana kajian ini dijalankan?

Untuk kajian ini, anda akan menyertai salah satu satu komponen kajian berikut: i) paras lignocaine dalam darah selama 3 jam, ii) perbandingan lignocaine-jarum mikro dengan pelekat EMLA.

i) Paras lignocaine dalam darah selama 3 jam

Jika anda adalah peserta kajian komponen ini, anda akan menerima tusukan vena untuk persampelan darah pada lawatan anda untuk rawatan susulan/ penjagaan harian di hospital. Seorang pegawai perubatan akan menjalankan kanulasi intravena pada bahagian belakang tangan kanan anda bagi tujuan untuk pengambilan sampel darah. Anda kemudiannya akan menerima ubat bius (lignocaine) melalui jarum mikro yang akan ditusuk pada permukaan kulit di belakang tangan kiri. Ini akan menyebabkan kesan bius setempat (rasa kebas) pada tangan anda yang ditetapkan dengan jarum mikro. Selepas itu, sampel darah (sekitar 3 mL setiap kali) akan diambil daripada kanula intravena pada enam tempoh masa yang tertentu (t= 0, 30, 60, 90, 120, dan 180 minit). Sampel darah ini kemudiannya akan dihantar ke makmal untuk menguji paras lignocaine dalam darah anda. Untuk komponen kajian ini, anda akan berada di klinik selama sekitar 3 jam, yang mana merupakan purata tempoh masa biasa setiap kunjungan ke klinik.

ii) Perbandingan lignocaine-jarum mikro dengan pelekat EMLA.

Jika anda merupakan peserta kajian komponen ini, anda akan terlebih dahulu menjalani pemeriksaan klinikal rutin pada hari kajian tersebut. Anda mungkin dipilih untuk menilai kadar kesakitan dasar anda dengan menggunakan suatu penyukat/pembaris-skor kesakitan (VAS) dan alat pemantauan kesakitan. Selepas itu, jarum mikro yang diresapi lignocaine atau pelekat EMLA, bergantung kepada kumpulan yang telah diperuntukkan kepada anda, akan dilekat pada permukaan belakang tangan anda yang telah dikenalpasti. Selepas 30 minit berlalu, prosedur tusukan vena akan dilaksanakan oleh pegawai perubatan terlatih. Anda kemudian diminta untuk menilai kadar kesakitan anda akibat tusukan vena atau kanulasi intravena tersebut dengan menggunakan skor kesakitan VAS. Selain itu, kabel elektrod mesin pemantauan kesakitan akan dilekatkan pada tapak tangan anda untuk pemantauan kesakitan. Anda akan dipantau selama satu jam selepas prosedur tersebut bagi mengenalpasti sama ada anda mengalami kesan sampingan yang disebabkan oleh tusukan jarum mikro-lignocaine.

Risiko kajian ini:

Penyelidikan ini membabitkan risiko yang minimum kepada peserta dan mempunyai kemungkinan yang rendah untuk menyebabkan kesan sampingan. Walaupun ubat bius diguna, kemungkinan sakit akibat tusukan jarum mahupun jarum mikro mungkin ada tetapi amat kurang. Darjah kesakitan adalah parameter utama dikaji. Ubat bius topikal lignocaine adalah biasa diguna dan kesan sampingan serius adalah amat kurang dilaporkan. Walau bagaimanapun, antara kesan sampingan yang mungkin berlaku akibat ubat bius lignocaine adalah merangkumi

- kepucatan,
- kemerahan,
- perubahan suhu di kulit kawasan aplikasi

Manakala untuk ubat EMLA, kesan sampingan kepada orang yang menghidapi kekurangan G6PD mungkin berisiko kejadian sejenak kenaikan paras methemoglobin dengan tanda kebiruan dan penurunan paras oksigen dalam darah. Sila beritahu pasukan penyelidik sebelum kajian jika kamu menghidapi kekurangan G6PD.
Risiko tambahan yang disebabkan oleh aplikasi jarum mikro, terutamanya bagi peserta yang memiliki kulit sensitif, berkemungkinan akan mengalami keradangan kulit ringan hingga sederhana seperti

- kemerahan,
- bengkak,
- gatal
- kulit melupuh

Kesan sampingan merupakan satu hasil kajian ini yang dikumpulkan. Sekiranya anda mengalami sebarang masalah kesan sampingan seperti di atas, kami akan memberi ubat kepada anda untuk rawatan mengurangkan kesan sampingan tersebut.

Faedah kajian ini:
Maklumat yang dikumpul dari kajian ini dapat menambahkan lagi pengetahuan perubatan mengenai keselamatan dan kecekapan penggunaan jarum mikro maltosa yang terlarut sebagai sistem penghantaran ejen bius setempat dan ia dapat membantu pesakit lain pada masa hadapan.

Adakah anda perlu menyertai kajian ini?
Penyertaan anda di dalam kajian ini adalah secara sukarela. Rawatan perubatan untuk anda tidak akan terjejas sekiranya anda memutuskan untuk tidak mengambil bahagian dalam kajian ini. Anda masih akan mendapat rawatan seperti biasa dan sama mengikut protokol pusat jagaan harian.
Sekiranya anda bersetuju untuk menyertai kajian ini, anda akan diminta untuk menandatangani "Borang Persetujuan Termaklum". Anda akan diberikan satu salinan borang persetujuan termaklum dan lembaran maklumat pesakit ini. Sekiranya anda bersetuju mengambil bahagian, anda tidak boleh memilih kumpulan rawatan yang akan diperuntukkan kepada anda. Akan tetapi, anda bebas untuk menolak diri dari kajian ini pada bila-bila masa tanpa sebarang alasan dan tanpa dikenakan penalti. Sekiranya anda memutuskan untuk menolak diri daripada kajian ini, anda perlu memaklumkan penyelidik kajian ini dan seterusnya tiada data baru akan diambil daripada anda.

Penyelidik juga mungkin mengeluarkan anda dari kajian di atas dengan sebab-sebab tertentu. Sekiranya keadaan ini berlaku, anda tidak akan menghilangkan hak anda sebagai pesakit dan akan terus menerima rawatan penjagaan seperti mana kebiasaannya.

Data & Kerahsiaan:
Kerahsiaan anda sebagai peserta akan dilindungi sepanjang masa penyelidikan. Data peribadi akan dirahsiakan. Oleh itu identiti anda akan disimpan secara sulit. Data yang dikumpulkan

dan dimasukkan dalam Borang Laporan Kes akan kekal menjadi hak milik UKM. Sekiranya terdapat penerbitan mengenai kajian ini, identiti anda akan kekal dirahsiakan.

Dengan menandatangani borang persetujuan yang dilampirkan sekali, anda (atau wakil anda yang boleh diterima secara sah, jika relevan) membenarkan akses untuk rekod kajian anda.

Bayaran & Pampasan:
Anda tidak perlu membayar ataupun akan menerima sebarang bayaran untuk penyertaan dalam kajian ini. Anda masih perlu membayar untuk perkhidmatan hospital seperti biasa.

Siapakah yang anda boleh hubungi untuk maklumat lanjut
Sekiranya anda mempunyai sebarang pertanyaan mengenai kajian penyelidikan tersebut atau hak-hak anda, sila hubungi

Penyelidik Utama: **Profesor Dr Cheah Fook Choe**
Jabatan Pediatrik
Pusat Perubatan Universiti Kebangsaan Malaysia
No. Telefon: 03-9145 5391

Penyelidik Bersama: **Profesor Dr Mae-Lynn Catherine Bastion**
Jabatan Oftalmologi
Pusat Perubatan Universiti Kebangsaan Malaysia
No. Telefon: 03-9145 5983

Dr Lam Chenshen
Jabatan Oftalmologi
Pusat Perubatan Universiti Kebangsaan Malaysia
No. Telefon: 03-8921 6520

Tandatangan
Untuk menyertai kajian ini, anda mesti menandatangani halaman tandatangan berserta tarikh. [LAMPIRAN A]

LAMPIRAN A

Borang Maklumat Pesakit/Subjek dan Persetujuan

(Halaman Tandatangan)

Tajuk Penyelidikan: **Penyerapan ubat lignocaine secara jarum mikro transdermal berbanding pelekat EMLA untuk kurangkan sakit sebelum prosedur tusukan vena bagi pesakit dewasa dalam persekitaran klinik.**

Nama penyelidik: Prof. Dr Cheah Fook Choe, ***Prof. Dr Mae-Lyn Catherine Bastion, Dr Lam Chen Shen***

Untuk menyertai kajian ini, anda mesti menandatangani halaman ini. Dengan menandatangani halaman ini, saya mengesahkan dengan yang berikut:

- Saya telah membaca dan memahami segala maklumat yang disediakan dalam Borang Maklumat Pesakit dan Borang Persetujuan ini, termasuk mana-mana maklumat berkaitan dengan risiko yang terlibat dalam kajian ini dan saya diberi masa yang cukup untuk mempertimbangkan kajian penyelidikan tersebut.
- Semua soalan saya telah dijawab sehingga sepuas-puas hati saya dengan jawapannya.
- Dengan ini, saya bersetuju untuk menyertai kajian penyelidikan ini secara sukarela, untuk mengikuti prosedur kajian dan memberi maklumat yang diperlukan kepada doktor, jururawat, atau ahli kakitangan lain, seperti yang diminta.
- Saya berbebas memilih untuk berhenti daripada kajian ini pada bila-bila masa.
- Saya telah menerima satu salinan Borang Maklumat Pesakit dan Borang Persetujuan untuk simpanan saya.

____________________<br>Nama Pesakit (Tulis atau taip)	____________________<br>Tandatangan dan Nombor Pesakit
____________________<br>Nombor K.P. Pesakit. (Baru)	
____________________<br>Tandatangan Pesakit<br>(Tambah waktu, jika berkenaan)	____________________<br>Tarikh(dd/MM/yy)
____________________<br>Nama Individu<br>Menjalankan Perbincangan Persetujuan (Tulis atau taip)	
____________________<br>Tandatangan Individu<br>Menjalankan Perbincangan Persetujuan	____________________<br>Tarikh (dd/MM/yy)
____________________<br>Nama dan tandatangan seksi	____________________<br>Tarikh (dd/MM/yy)

<u>Nota:</u> i) Semua subjek/pesakit yang terlibat dalam kajian ini tidak akan dilindungi oleh insurans.

APPENDIX B: PATIENT INFORMATION SHEET AND CONSENT FORM (ENGLISH VERSION)

PATIENT INFORMATION SHEET

Research Title: Transdermal microneedle lignocaine delivery versus EMLA patch for topical analgesia before venepuncture procedure to adults in a clinic setting

Introduction:
You are invited to participate in a clinical research study. Before participating in this study, it is crucial that you read thoroughly and understand the information provided in this sheet, whereby the study will be verbally explained to you, and you will be allowed to ask questions. After you are adequately satisfied that you understand this study and you wish to take part or continue to participate in this study, you must sign this informed consent form. You will be given a copy of these patient information sheet and consent forms to take home with you.

Purpose of Study:
Vein-puncturing procedures elicits much pain and is regarded as the most frequently traumatic experience encountered among patients in the regular practice of the health care settings. Such pain may be reduced by applying topical anaesthetic drugs. For example, lignocaine cream is a non-invasive and convenient approach of administering anaesthesia to the body skin surface. The usual recommended duration of time taken for lignocaine cream to work is around 30 minutes. However, in a busy clinical setting, the time is often shortened to 15 minutes for a slight anaesthetic effect. As such, a transdermal drug delivery system (TDDS), the microneedle, has been introduced as an alternative to enhance the delivery of topical anaesthesia by puncturing the skin at a very superficial level to create multiple tiny tracts for faster action of anaesthetic drug absorption. The microneedle patch is a prototype device that is still being tested and not registered. It contains multiple micron-sized needles made of sugar (maltose) that will dissolve into the skin and subsequently achieve the objective of delivering the anaesthetic drugs. As such, our current research study aims to determine the safety and tolerability of lignocaine-embedded microneedle patch for patients requiring frequent venepuncture or intravenous (IV) cannulation. Besides, this research also aims to obtain preliminary information on the efficacy of lignocaine-embedded microneedle in reducing pain associated with venepuncture or intravenous cannulation.

What will the study involve?
For this research, you will be participating in either one of the following components of the trial: i) blood levels of lignocaine over 3hours or ii) lignocaine-microneedle versus EMLA patch. It is emphasised that you do not get to choose which group you will be in if you agree to participate. We use a selection system that the project investigators are abiding to.

i) Blood level of drug study over 3 hours

In this study, you are will undergo a venepuncture for blood collection during an outpatient visit at the Ophthalmology Clinic, HCTM. A medical doctor will perform an intravenous cannulation on the back of your right hands (dorsal side) that is intended for blood collection. Subsequently, you will be receiving a topical anaesthetic administration via lignocaine-embedded microneedle which will be applied on the skin surface of the back of your left hand. This will result in the numbness to that particular region of the hand. A small amount of blood (about 3 mLs each time) samples will then be collected from the cannula at six time points (time, t= 0, 30, 60, 90, 120, and 180 minutes) which will be sent to the laboratory for determining the level of lignocaine in your blood. In total, you are expected to be at the clinic for 3 hours, which is the average time taken for a normal visit to the eye clinic.

ii) Lignocaine-microneedle versus EMLA patch

If you are selected as a participant of this part of the research, you will first undergo a routine clinical examination on the trial day. You may be chosen to rate your baseline pain score using a ruler-styled scoring system (VAS) and a pain monitoring device. The lignocaine-embedded microneedle or EMLA patch, depending on which group you will be allotted to, will be introduced on the identified surface of the back of your hand. After 30 minutes, venepuncture or intravenous cannulation will be performed by a trained medical officer. You will be then asked to evaluate the degree of your pain using the VAS score. You will also have a pain monitoring device attached to your palm for measurement purposes. You will then be monitored for one hour following the procedure to identify whether you experience any adverse effects associated with the lignocaine-microneedle application.

Risks:

This investigation possesses minimal risk to participants and is unlikely to cause side effects. Although topical skin anaesthetics are applied, you may still feel some pain from the needle/s, although this will be much reduced. Topical lignocaine is a widely used drug and little adverse reactions have been reported. Nevertheless, the possible side effects reported from the lignocaine application include:

- pallor
- redness
- alterations in temperature sensation over the application area

With regards to EMLA patch, special precautions are advised in G6PD deficient individuals who may be at increased risk for developing symptoms as a result of methaemoglobin rise in blood causing temporarily a bluish tinge to the skin and decreased oxygen in the blood. If you are G6PD deficient, please inform us about this condition prior to the study.
Additional risks associated with microneedle application might include possible mild-to-moderate irritation, especially in those with sensitive skin, such as

- redness
- swelling
- itching
- blistering

Adverse side-effects reporting is part of the outcomes of this study, but If you encounter any problems or side effects experienced during the study, we will be giving you medications to reduce the side effects accordingly.

Benefits:

The information collected from this clinical study may contribute to the advancement of medical knowledge on the safety and efficacy of dissolving microneedle in delivering local anaesthetic agents, which will benefit patients in the future.

Do you have to take part?

Your participation in this study is absolute voluntarily. Your medical care will not be affected if you decide not to participate in this study. You will still have the usual standard of care according to the day-care protocol.

If you agree to participate, you will be asked to sign the "Informed Consent Form". You will be given a copy of the informed consent form and this patient information sheet. Should you decide to participate, you cannot decide which group you will be assigned to, but you are still free to withdraw from the study at any time without giving a reason or penalty. If you decide to cease from participating in this study, you must inform your study investigator and no new data will be further collected from you. The researcher may also remove your participation from the study for various reasons. In this event, you will not lose your rights as a patient and will still receive the usual standard of care.

Data & Confidentiality:

Participant's confidentiality will be maintained throughout the investigation. Your personal data well be anonymized as your identity be always kept confidential. Data collected and entered into the Case Report Form will remain as the governed property of UKM. In the event of any publication generated from this study, your identity will be remained confidential to the public.

By signing the Informed Consent Form attached, you (or your legally acceptable representative, if relevant) are authorizing such access to your study records.

Payment and compensation:

You do not have to pay, nor will you be paid to participate in this study. You do have to pay for the usual hospital charges.

Whom can I ask about the study?

If you have any questions about this study or your rights, please contact:

**Principal Investigator:**	**Prof. Dr Cheah Fook Choe**<br>**Department of Paediatrics**<br>**UKM Medical Centre**<br>**Phone Number : 03-9145 5391**
**Co-investigator**	**Prof Dr Mae-Lyn Catherine Bastion**<br>**Department of Ophthalmology**<br>**UKM Medical Centre**<br>**Phone Number: 03-9145 5983**
	**Dr Lam Chenshen**<br>**Department of Ophthalmology**<br>**UKM Medical Centre**<br>**No. Telefon: 03-8921 6520**

Signatures

To be selected into this study, you must sign and date the signature page [ATTACHMENT A]

ATTACHMENT A

Patient/Subject Information and Consent Form

(Signature Page)

Research Title: **Transdermal microneedle lignocaine delivery versus EMLA patch for topical analgesia before venepuncture procedure to adults in a clinic setting**

Researcher's Name: Prof. Dr Cheah Fook Choe, ***Prof. Dr Mae-Lyn Catherine Bastion, Dr Lam Chen Shen***

To become a part of this study, you must sign this page. By signing this page, I amconfirming the following:

- I have read and understand all the information in this Patient Information Sheet and Consent Form, including any information regarding the risk stated in this study and I, have given sufficient time to consider about this study.
- All of my questions have been answered to my satisfaction.
- Hereby, I voluntarily agree to be part of this research study, to follow the study procedures, and to provide necessary information to the doctor, nurses, or other staff members, as requested.
- I may freely choose to stop being a part of this study at any time.
- I have received a copy of this Patient Information Sheet and Consent Form to keep for myself.

__________________________________ ____________________

Patient Name (Print or type) Patient Initials and Number

____

Patient I.C No. (New)

__________________________________ ____________________

Signature of Patient Date (dd/MM/yy)
(Add time if applicable)

____

Name of Individual
Conducting Consent Discussion (Print or Type)

__________________________________ ____________________

Signature of Individual Date (dd/MM/yy)
Conducting Consent Discussion

__________________________________ ____________________

Name & Signature of Witness Date (dd/MM/yy)

Note: i) All subject/patients who are involved in this study will not be covered by insurance

APPENDIX C: CASE REPORT FORM (CRF)

CASE REPORT FORM

Research title: Transdermal microneedle lignocaine delivery versus EMLA patch for topical analgesia before venepuncture procedure to adults in a clinic setting

Research Investigators: Professors Dr Cheah Fook Choe, Mae-Lynn Catherine Bastion, Dr Lam CS.

Sticker Pesakit

ID Subjek: *Subject ID* ☐☐☐☐☐

Tarikh: *Date* ☐☐ / ☐☐ / ☐☐
Hari *Day* / Bulan *Month* / Tahun *Year*

Kod Rawak:
Randomisation code

A. MAKLUMAT SUBJEK
SUBJECT'S DETAIL

Nama Subjek : ____________________
Subject's name

Usia / *Age* : __________ tahun / *years* ________ bulan / *months*

No. Pend / *RN* : ____________________

No. telefon : Telefon bimbit : ____________________
Telephone no. *Handphone*

Telefon rumah : ____________________
Home

Tarikh lahir : ________ / ________ / ________
Date of birth

Tarikh keizinan: ________ / ________ / ________
Date of consent

Jantina : ☐ Lelaki *Male* ☐ Perempuan *Female*
Gender

Kaum : ☐ Melayu *Malay* ☐ Cina *Chinese* ☐ India *Indian*
Ethnicity

Lain-lain, silanyatakan: ____________________

Others, please specify

B. SEJARAH PENYAKIT KELUARGA
FAMILY HISTORY OF ILLNESS

Adakah sesiapa ahli keluarga yang dijangkit penyakit yang ketara dalam sejarah penyakit mereka:
Has any of the family members having any significant disease in their history of illness.

**Darjahpertama** *First degree*	**Ya**	**Tidak**
Bapa *Father*	☐	☐
Ibu *Mother*	☐	☐
Adik-beradik *Siblings*	☐	☐

Lain-lain: ____________________________
Others

Jika Ya, sila nyatakan jenis penyakit yang terlibat dan umur ketika diagnosis: ____________________________

If Yes, please specify the types of diseases and age at diagnosis: ____________________________

____

C. KAJIAN SEJARAH PERUBATAN / PENGAMBILAN UBAT BERKAITAN
REVIEW OF MEDICAL HISTORY / MEDICATIONS CHECKLIST

Sejarah perubatan yang berkaitan:
Relevant medical history

Kencing Manis : ☐
Diabetes

Darah Tinggi : ☐
Hypertension

Penyakit Buah Pinggang : ☐
Kidney disease

Penyakit Autoimun : ☐
Autoimmune disease

Penyakit Hati : ☐
Hepatic diseases

Lain-lain : ____________________
Others ____________________
____

Pengambilan ubat berkaitan (sila nyatakan nama ubat, tujuan perubatan, dos dan cara pengambilan)
Medications (please specify the name, indications, dosage and administration frequency)

Nama	Tujuan	Dos	Cara pengambilan

*Senarai semak ubat-ubat
Medication checklist

Erythromycin ☐
Ciprofloxacin ☐
Amiodarone ☐
Beta-adrenergic blockers ☐
(timolol eyedrops, metoprolol etc)

Adakah pesakit pernah mengalami alahan/ kesan sampingan yang teruk daripada mana-mana ubat?
Has subject ever experienced any allergy or adverse event from any medications?

Ya * ☐ Tidak ☐
Yes *No*

***Jika '**Ya**', sila nyatakan jenis ubat yang terlibat: ______________________
*If **yes**, please specify the type of medication*

Sila nyatakan jenis diagnosis **MATA** dan pembedahan **MATA** yang dirancangkan:
Please specify the type of ocular diagnosis and the planned operation

Diagnosis: ______________________ Tarikh: ______________

Mata Kiri ☐ Kanan ☐

Pembedahan / *Surgery*:

Phacoemulsification: ☐
ECCE: ☐
Trabeculectomy: ☐
Glaucoma drainage device: ☐
Pars plana vitrectomy: ☐
Corneal transplant: ☐
Lain-lain/ *Others*: ______________________
____
____

D. DATA KLINIKAL
CLINICAL DATA

Pengukuran antropometri:
Anthropometric measurement

1. Berat : kg
   Weight

2. Tinggi : cm
   Height

3. Indeks Jisim Tubuh (BMI) : kg/m^2
   Body mass index

Tanda-tanda vital
Vital signs

			**Untuk kegunaan jika diperlukan**<br>*To be applied if needed*	
	Unit<br>*Unit*	Lawatan klinik<br>*Clinic visit*	**PK Study**	
		Tarikh/ Date:<br>__ / __ / __	**Tarikh/Date:**<br>**__ / __ / __**	**Blood Sample (Y/N)**<br>**Volume (mL)**
Tekanan Sistolik<br>*Systolic pressure*	mmHg		Time: 0 min	
Tekanan Diastolik<br>*Diastolic pressure*	mmHg		Time: 30 mins	
Nadi<br>*Heart rate*	bpm		Time: 60 mins	
Paras gula<br>*Dextrostix*	mmol/L		Time: 90 mins	
Skala kesakitan (VAS)<br>*Pain scale (VAS)*			Time: 120 mins	
Skala Kesakitan (Pain Monitor)<br>*Pain score (Pain Monitor)*			Time: 180 mins	
			Samples sent to Jabatan Kimia	Date:<br><br>Time:
Tandatangan dan nama ringkas pemeriksa<br>*Initials and name of examiner*				

LOG PENJEJEKAN KESAN SAMPINGAN / ADVERSE EFFECTS TRACKING LOG

No.	Date reported	Adverse event description	Start date	End date	Ongoing (Yes or No)	Outcome[1]	Severity / grade[2]	Serious (Yes or No)	AE treatment[3]	Expected (Yes or No)	Intervention Attribution / Relatedness[4]

Scales:

**Outcome[1]**	**Severity / grade[2]**	**AE treatment[3]**	**Intervention Attribution / Relatedness[4]**
0- Fatal	0- Mild	0- None	0- Definite
1- Not recovered / Not resolved	1- Moderate	1- Medication(s)	1- Probable
2- Recovered w/sequelae	2- Severe	2- Medication TX	2- Possible
3- Recovered w/o sequelae	3- Life-threatening		3- Unrelated
4- Recovering / Resolving	4- Death / Fatal		4- Not applicable (did not receive intervention)

Verified by (Prior to data entry):

Signature:

Name:
Date:
