# Supplementary material for: The efficacy and safety of lignocaine-embedded dissolvable microneedle versus EMLA for topical analgesia in adults undergoing venepuncture: A single-centre, parallel-group, double-blind randomised clinical trial protocol in a tertiary care setting
Source: PLoS One. 2025 Nov 4;20(11):e0335932. doi: 10.1371/journal.pone.0335932 (PMC12585024; doi:10.1371/journal.pone.0335932)
Supplement: Attachment — Submitted filename: Response to Reviewers (PONE-D-25-38618R1).docx [file pone.0335932.s005.docx]

**Appendix A**

**A) Reviewer 1: Point by Point Responses**

**1) Comment 1:** Protocol prerequisites – The manuscript states that Phase I recruitment began on 17 February 2025 and is expected to finish by 16 October 2025, with Phase II due to start in November 2025. Because participants are already being enrolled, please confirm that no outcome data have been analysed and explain how the manuscript continues to qualify as a pre-result “Study Protocol”.

**i) Author’s Response**

We sincerely thank the reviewer for raising this point. We confirm that no outcome data have been analysed. No interim, exploratory, or final analyses of primary or secondary endpoints have been conducted. No unblinding has occurred. Monitoring has been limited to routine trial conduct checks such as screening logs, accrual counts, and safety oversight, which do not involve outcome analysis.

The submission qualifies as a pre-results Study Protocol because journals that publish protocols define “pre-results” by the absence of analysed outcomes and by the fact that recruitment or data collection is not yet complete. PLOS ONE’s reviewer guidance states that Study Protocols should not have generated results and recruitment or data collection should not be complete, while allowing feasibility or pilot information only when necessary for proof of principle [1]. Trials specifies that protocols are generally considered for proposed or ongoing studies before recruitment completes and may be considered up to before last patient last visit provided the manuscript clearly states the status at submission and explains any delay [2]. BMJ Open advises that studies that have not completed recruitment or baseline data collection should be submitted as study protocols [3]. These policies align with SPIRIT 2025, which governs the content and transparency of trial protocols and does not require that recruitment be entirely prospective for the protocol article itself, provided that all prespecified plans, governance, and reporting elements are fully described [4]. Together, these policies mean that a protocol can be submitted and published while recruitment is underway, so long as no outcome data have been analysed and the manuscript transparently reports trial status. [1-4]

For completeness, we also confirm prospective registration of our trial protocol and adherence to accepted standards. The trial was prospectively registered in a ICMJE-recognised public registry before the first participant was enrolled, in line with ICMJE policy and WHO standards on prospective registration. The registry identifier and registration date are reported in the manuscript. [5, 6]

To address your request for clarity within the manuscript, we will add the following statement in the “Trial status” section and in the cover letter.

**ii) Manuscript changes (In Methodology, Trial Status Subsection)**

“At the time of submission, no outcome data have been analysed and no unblinding has occurred. This article reports the pre-results trial protocol in accordance with SPIRIT 2025 guideline [28].”

**iii) References Used in Responses**

1. PLOS ONE. Reviewer guidelines. 2025.

2. Trials. Study protocols policy update. 30 January 2020. Also see Trials “Study protocol” submission guidelines, current version.

3. BMJ Open. Authors page. Guidance on protocol submissions for studies that have not completed recruitment or baseline data collection.

4. Hróbjartsson A, Boutron I, Hopewell S, Moher D, Schulz KF, Collins GS, et al. SPIRIT 2025 explanation and elaboration. BMJ. 2025;389:e081660. doi:10.1136/bmj-2024-081660.

5. International Committee of Medical Journal Editors. Clinical trial registration policy and FAQs.

6. World Health Organization. International standards for clinical trial registries. 2018.

_____________________________________________________________________

**2) Reviewer’s Comment 2:** Rationale & research questions – The introduction clearly identifies the gap (few adult data on microneedle anaesthesia for venepuncture) and proposes LEMAP as a solution. However, explicit directional hypotheses and a patient-centred minimal clinically important difference (MCID) are not stated. Please add these to sharpen the objectives.

**i) Author’s Response**

Thank you for the suggestion. We have added explicit directional hypotheses and a patient-centred MCID to sharpen the objectives. The hypotheses state that LEMAP will reduce pain more than the EMLA sham patch on both co-primary outcomes (VAS and SCAI). We also prespecify a 10 mm MCID on the 0–100 mm VAS, which aligns with the value used for Phase II sample size and will guide interpretation. These additions appear at the end of the introduction section.

**ii) Manuscript changes (In Introduction)**

“We hypothesise that, at one minute after venepuncture, participants receiving LEMAP will report lower pain than those receiving the EMLA sham patch on both co-primary outcomes. Specifically, the mean VAS will be lower with LEMAP than with control, and the mean SCAI will be lower with LEMAP than with control. We prespecify a patient-centred minimal clinically important difference (MCID) of 10 mm on the 0-100 mm VAS scale to guide interpretation and to underpin the Phase II sample size calculation.” (**Page 7, Lines 114-120**)

_____________________________________________________________________

**3) Reviewer’s Comment 3 (specifically point 3.2):** Sample size – The Phase II calculation assumes a “1-point VAS difference” with SD = 2 and Cohen’s d = 0.5. Given that VAS is defined as 0–100 mm (not 0–10 points) elsewhere in the manuscript, the target effect appears to be ~10 mm. This is at the lower boundary of published MCIDs (≈9–13 mm) for acute pain; please justify or consider re-powering for ≥13–15 mm.

**i) Author’s Response**

We thank the reviewer for this helpful comment. We agree that all pain outcomes should be expressed on the 0-100 mm VAS scale. We have corrected the units in the Phase II sample size paragraph and now state a 10 mm target difference with SD 20 mm, which corresponds to the same standardized effect size (Cohen’s d = 0.5) used previously. A 10 mm difference is consistent with the lower end of published minimal clinically important differences (MCIDs) for acute pain and has been repeatedly supported in emergency and perioperative studies [1-5]. Todd et al. reported about 13 mm as the smallest important change adults can perceive on a 100 mm VAS [1], with similar findings from Gallagher et al [2]. Kelly reported an MCID of 9 mm VAS in an emergency setting [3], and a postoperative study by Myles et al. triangulated an MCID of approximately 10 mm [4]. A systematic review found threshold-based MCIDs with a median of 10 mm VAS score across acute pain studies, based on the threshold method for absolute VAS change [5].

On this basis, 10 mm is a reasonable and conservative value to power a confirmatory trial. Re-powering to ≥13-15 mm would assume a larger effect than is necessary and could reduce the required sample size while increasing the risk of type II error if the true effect is closer to 10 mm. We therefore retain 10 mm (SD 20 mm) and have clarified the units in the manuscript with citations to the MCID literature.

**ii) Manuscript changes (In Methodology, Sample Size Calculation Subsection, Under Phase II: Efficacy Study Sub-subsection)**

“We consider a 10-mm point VAS difference on the 0-100 mm VAS scale between the intervention group as the MCID for acute procedural pain, based on the threshold approach reported in Olsen and colleagues in their systematic review [12] and to reduce the probability of type II error if the true treatment effect is actually closer to this conservative choice of MCID.” (**Page 13, Lines 173-176**)

**iii) References Used in Responses**

1) Todd KH, Funk KG, Funk JP, Bonacci R. Clinical significance of reported changes in pain severity. Ann Emerg Med. 1996;27(4):485-9.

2) Gallagher EJ, Liebman M, Bijur PE. Prospective validation of clinically important changes in pain severity measured on a visual analog scale. Ann Emerg Med. 2001;38(6):633-8.

3) Kelly AM. The minimum clinically significant difference in visual analogue scale pain score does not differ with severity of pain. Emerg Med J. 2001;18(3):205-7.

4) Myles PS, Myles DB, Galagher W, et al. Minimal clinically important difference for three quality of recovery scales after general anaesthesia. Br J Anaesth. 2017;118(3):424-9

5) Olsen MF, Bjerre E, Hansen MD, et al. Pain relief that matters to patients: Systematic review of MCID for acute pain measured on VAS/NRS. BMC Med. 2017;15:35. (**reference 12 in the main manuscript text**)

___________________________________________________________________________

**4)** **Reviewer’s Comment 4:** Blinding – LEMAP dissolves after 2 min while the EMLA sham stays occluded for 30 min, which may unmask allocation to clinicians. Describe tactile/visual masking steps or harmonise application times.

**i) Author’s Response**

Thank you for the helpful comment. We confirm that the LEMAP’s maltose microneedle matrix dissolves within about 2 minutes. The patch will be nevertheless kept in place for 30 minutes to standardise application time between the LEMAP and control (sham patch) groups, preserve blinding (masking) between study arms, and allow sufficient time for dermal uptake of lignocaine.

**ii) Manuscript changes (In Methodology, Trial Status Subsection)**

“Although LEMAP fully dissolves within approximately 2 minutes, the procedurist will keep the patch in place for 30 minutes to standardise application time, maintain blinding, and ensure adequate dermal uptake before cannulation.” (**Pages 21-22, Lines 384-387**)

_____________________________________________________________________

**5) Reviewer’s Comment 5:** Methodological detail for replication – Device fabrication steps are outlined, but key specifications (needle density, tip radius, residual lignocaine after dissolution) are missing. Likewise, SCAI sensor placement/calibration and VAS ruler calibration should be elaborated to enable replication.

**i) Author’s Response**

Thank you for your highly relevant and cogent suggestions. We have added the missing device specifications and clarified our dosing estimate for lignocaine. We now state that the microneedle tip density is 225 needles per cm², tip radius 2 μm and base radius 50 μm. We explain that residual lignocaine is typically about 20 percent of the initial load post-application, so about 80 percent is potentially delivered, and we show how this is approximated using the conical volume formula.

We have also added explicit procedures for SCAI sensor placement and calibration and for VAS ruler calibration to enable replication. These insertions are placed in the Intervention Characteristics and Trial Outcomes sections of the manuscript.

**ii) Manuscript changes (In Several Sections)**

**a) Methodological Section (In Physical Descriptions of the Microneedle Array Patch Subsection)**

“Each LEMAP contains 225 microneedles per cm². The tip radius is 2 μm and the base radius is 50 μm. Lignocaine load is assumed to be uniformly distributed within each microneedle. The total lignocaine volumes per microneedle before application and after application are approximated using the conical volume formulae, $V= \frac{1}{3}\pi r^{2}h$and $V= \frac{1}{3}\pi r^{2}h^{*},$ with r as the base radius, h as the microneedle height before LEMAP application and $h^{*}$ after LEMAP application. The maltose matrix volume was considered as negligible for lignocaine dose calculation. Post-application, about 20 percent of the initial lignocaine typically remains undissolved within the microneedle, so approximately 80 percent is potentially delivered to the skin. This geometric approximation is used only to standardise reporting of dose delivery since pharmacokinetic sampling in our Phase I trial remains the determinant of systemic exposure.” (**Pages 16-17, Lines 255-264**)

**b) Methodological Section (In Trial Outcomes Subsection, Under Primary Endpoint) - For VAS ruler calibration**

“For VAS instrument calibration and standardisation, we will use a 0-100 mm slider VAS ruler of fixed length. Before first use and weekly thereafter, the research team will verify the physical scale length against a well-calibrated and standardized steel ruler and confirm the 0 mm and 100 mm markings on the VAS ruler align 0 mm and 100 mm markings on the reference ruler. VAS ruler that fails this check will be replaced. The participant will set the slider unaided, the trained outcome assessor (medical officer) will read the value from the reverse side.” (**Page 22-23, Lines 405-411**)

**b) Methodological Section (In Trial Outcomes Subsection, Under Primary Endpoint) - For SCAI Sensor Placement and Calibration**

“The skin conductance algesimeter index (SCAI), measured in microSiemens per second (μS/s), will be obtained using PainMonitor™ (Med-Storm Innovation AS, Oslo, Norway) (Figure 7). The device consists of a sensor will be attached to the hypothenar eminence of the hand of the limb not subjected to venepuncture. The skin will be cleaned with alcohol swab and allowed to air dry before PainMonitor™’s sensor is attached. The single-use sensor will be applied with firm, even contact according to the manufacturer’s instructions. The wrist and hand will be supported to minimise motion. The same limb and site will be used across participants whenever feasible.” (**Page 23, Lines 412-419**)

“For SCAI calibration, the operator will perform the PainMonitor™’s built-in quality check, confirm stable baseline conductance and acceptable signal quality and document ambient temperature and obvious sources of electrical or motion artefact. A baseline of at least 60 seconds will be recorded before venepuncture with the participant resting quietly. The post-venepuncture SCAI value obtained at one minute will be the primary time point, as prespecified above. If artefact is detected during this 60-second period, the operator will mark the trace and repeat a one-minute acquisition once the artefact resolves, without revealing treatment allocation. The same device model will be used throughout and operators will follow a standard operating procedure that includes periodic retraining and inter-operator checks.” (**Page 23, Lines 424-432**)

_____________________________________________________________________

**6) Reviewer’s Comment 6a:** Primary comparisons use t/Mann-Whitney tests, then linear regression with covariate selection by multiple strategies. Mixing stepwise (AIC/BIC), purposeful selection and clinical judgement is overly flexible and can inflate false-positive rates. Specify a single, preferably pre-defined strategy—e.g., include only clinically justified pre-specified covariates plus any baseline imbalance >10 %.

**i) Author’s Response**

Thank you for your comment on this pertinent point about the inflated type I (false-positive) error rate from mixing variable selection strategies. We agree and have simplified the analysis plan to a single, pre-defined approach. The primary multiple linear regression (MLR) will adjust for a fixed set of clinically justified covariates (baseline VAS, baseline SCAI, age, sex, BMI). No stepwise/AIC/BIC/purposeful selection will be used. To address any meaningful baseline imbalance, we will add any other measured baseline covariate that shows a standardized difference of more than 10% between arms to the model under a pre-specified rule. We will report baseline balance using standardized differences and will not use significance testing of baseline characteristics to drive model specification. This aligns with the TRIPOD guideline to avoid data-driven selection and to pre-specify predictors to limit overfitting and enhance reproducibility [1].

**ii) Manuscript changes (In Methodology, Statistical Analysis Plan Subsection)**

“To control and adjust the effects of clinically-justified pre-specified covariates (confounders) such as baseline pain scores (baseline VAS and baseline SCAI), body mass index (BMI), gender, multiple linear regression (MLR) analysis will be used. We will not use automated or data-driven variable selection (no stepwise/AIC/BIC or purposeful selection). In addition, if any other measured baseline covariate shows a standardised difference >10% between arms, it will be added under this pre-specified rule to mitigate chance imbalance. Baseline balance will be summarized using standardized differences, but significance testing of baseline characteristics will not guide model specification.” (**Pages 29-30, Lines 575-582**)

**iii) Reference used in Response**

1) Moons KG, Altman DG, Reitsma JB, Ioannidis JP, Macaskill P, Steyerberg EW, Vickers AJ, Ransohoff DF, Collins GS. Transparent Reporting of a multivariable prediction model for Individual Prognosis or Diagnosis (TRIPOD): explanation and elaboration. Ann Intern Med. 2015;162(1):W1-73. doi: 10.7326/M14-0698

_____________________________________________________________________

**7) Reviewer’s Comment 6b:** Penalised methods (LASSO) are proposed as a sensitivity analysis; clarify whether shrinkage estimates will be purely exploratory or could alter primary inference.

**i) Author’s Response**

Thank you for your comment. Penalised methods used in the sensitivity analysis are for exploratory purposes only. They are included to check model stability and will not change the trial’s efficacy conclusions or inference. Primary inference will come from the prespecified covariate-adjusted multiple linear regression. If model diagnostics show important assumption violations that are not resolved by transformation, we will use the prespecified alternatives of median quantile regression with the same covariates and a generalised additive model (GAM) with penalised splines for continuous covariates, while keeping the treatment term parametric. In all penalised models, the treatment indicator will be forced into the model and not penalised. Outputs from penalised analyses will be reported descriptively.

**ii) Manuscript changes (In Methodology, Statistical Analysis Plan Subsection)**

“For penalised regression methods (e.g. LASSO), they will be used for exploratory purposes only to check model stability and will not change the primary efficacy inference. The treatment indicator will be included in all penalised models and not penalised and the findings will be reported descriptively.” (**Page 31, Lines 613-616**)

_____________________________________________________________________

**8) Reviewer’s Comment 6c:** Two co-primary endpoints (VAS, SCAI) require an α-adjustment, not mentioned.

**i) Author’s Response**

Thank you for comments on the multiplicity issue with our co-primary endpoints. Our protocol and registry specify two co-primary endpoints and no secondary endpoints. We now make the error-control explicit. The study will be declared positive only if both co-primary hypotheses are significant at two-sided α=0.05. This conjunctive co-primary framework controls the family-wise Type I error without splitting α and is consistent with regulatory guidance on multiple endpoints [1]. For instance, in Section III.A.1 in the US-FDA guideline [1], it was stated that (quote verbatim) “**The second is when the determination of effectiveness depends on success on all primary endpoints, when there are two or more primary endpoints. In this setting, there are no multiplicity issues related to primary endpoints, as there is only one path that leads to a successful outcome for the trial and therefore, no concern with Type I error rate inflation**.” Besides, in Section III.C.1, the US-FDA guideline further stated this “**As discussed in section III.B., there is no multiplicity problem when the study is designed to** **demonstrate efficacy on all of the separate endpoints**”. Hence, we a trial success is contingent upon achieving statistical significance on both co-primary endpoints, hence no alpha adjustment is required.

To clarify our point further, we have added several sentences in our revised manuscript.

**ii) Manuscript changes (In Several Sections)**

**a) Methodology Section (In Trial Endpoint Subsection, Under Primary Endpoint Sub-subsection)**

“The Phase II trial has two co-primary endpoints.” (**Page 2, Line 398**)

**b) Methodology Section (In Statistical Analysis Plan Subsection)**

“Family-wise two-sided α of 0.05 will be controlled by requiring significance on both co-primary endpoints (VAS and SCAI). Each endpoint will be tested at two-sided α of 0.05. This conjunctive rule provides strong control of the Type I error in line with the US Food and Drug Administration’s (US-FDA) guidance on multiple endpoints for confirmatory trials [28].” (**Page 29, Lines 570-574**)

**iii) References Used in Responses**

1) U.S. Food and Drug Administration. Multiple endpoints in clinical trials: Guidance for industry. Silver Spring MD: FDA; 2022 Oct. Date of Access: 12th Sept 2025. [cited as reference 28 in the main text of the manuscript]

_____________________________________________________________________

**9) Reviewer’s Comment 6d:** Missing-data strategy. This is a single-visit study in which VAS and SCAI are recorded immediately after venepuncture, so missing outcome data should be rare. Unless you expect > 5 % missingness, multiple imputation may add unnecessary complexity. If you keep it, please pre-specify: (i) the algorithm (e.g., MICE with predictive mean matching), (ii) the number of imputations (≥ 20), (iii) the auxiliary variables to be included, and (iv) the δ values or shift parameters for any pattern-mixture sensitivity analyses.

**i) Author’s Response**

We thank the reviewer for this insightful comment. We agree that outcome missingness is expected to be low in a single-visit design. However, we maintain our multiple imputation strategy to uphold rigor and ITT analysis principles. Missing data can still occur even in one-visit studies, and ignoring them or using complete-case analysis could introduce bias [1]. We provide below a detailed justification for retaining multiple imputation, clarify our imputation methods, and describe our planned pattern-mixture sensitivity analysis addressing potential MNAR (missing not at random) scenarios.

**Justification for Multiple Imputation (MI)**

Even with an immediate post-procedure outcome, there are realistic scenarios where >5% missingness could occur. We believe proactively planning for MI is warranted, for the following reasons:

- **Participant unavailability:** Participants might withdraw or be unable to complete the post-venepuncture pain assessments (e.g., due to vasovagal syncope, acute distress, or early withdrawal). Such unexpected events could lead to missing VAS/SCAI outcomes even in a single-visit setting.
- **Technical or procedural issues:** The SCAI measurement device could malfunction or yield no reading for some patients, and VAS forms might occasionally be misplaced or not completed. These practical issues, although infrequent, can lead to missing outcome data.
- **Anticipated attrition:** Our sample size calculation already accounted for up to ~10% dropout/attrition, which suggests that missing outcome data >5% is plausible. If a participant drops out after randomization (even if the intervention was applied), their outcome might be missing. We thus plan to include such cases via imputation to preserve the intention-to-treat population.

Given these considerations, missing data, albeit limited, “are unintended, but unfortunately inevitable in clinical trials” [1]. Even a small amount of missing outcomes can potentially bias results if those missing differ systematically from those observed. It is for this reason that multiple imputation is widely recommended for handling missing data in clinical trials, provided the Missing At Random assumption is reasonable [2]. In line with best practices, we pre-specified MI to ensure all randomized participants are analysed (adhering to ITT) and to maximize statistical power and validity of our estimates. If missingness indeed turns out very low, MI will still yield nearly identical results to a complete-case analysis, but it provides reassurance that any potential bias is addressed proactively. This conservative approach follows expert guidance that emphasizes proper planning for missing data in confirmatory trials [1-2]

**Multiple Imputation Method Details**

We confirm that we will retain the multiple imputation strategy and provide the following specifics, as requested:

a) **Imputation algorithm:** We will use multiple imputation by chained equations (MICE) with predictive mean matching for our continuous endpoints (VAS and SCAI). This method is chosen because MICE is a flexible, practical approach for handling missing data [2] and predictive mean matching in particular will ensure imputed pain scores are plausible (bounded by observed values and maintaining the distributions).

b) **Number of imputations:** We plan to generate 20 imputed datasets initially. This total number of imputations is above the common rule-of-thumb (which suggests using at least as many imputations as the percentage of incomplete cases) and should be ample given our expected low missing rate. If necessary, for instance, if diagnostics indicate high variability in estimates, we will increase the number of imputations to ensure stability of results. Using 20+ imputations balances efficiency with precision of the pooled estimates [2].

c) **Auxiliary variables**: Our imputation model will include baseline VAS and baseline SCAI scores as auxiliary predictors (in addition to treatment group and other relevant variables). The rationale is that baseline pain and baseline SCAI readings are strong predictors of post-venepuncture pain outcomes and may also be related to any propensity to have missing data. Including these auxiliary covariates improves the accuracy of the imputation and helps to satisfy the Missing At Random (MAR) assumption (by accounting for variables that might influence missingness). In practical terms, this means the imputation procedure will “borrow strength” from patients’ baseline pain levels to inform plausible values for missing outcome data, thereby yielding more reliable and unbiased estimates [2].

These implementation details have been added to our Statistical Analysis Plan to assure readers that the multiple imputation is conducted with appropriate methodology (MICE with predictive mean matching) and parameters (number of imputations and model covariates) in accordance with standard guidance [2]. Besides, by clarifying this, we aim to address the reviewer’s concern that the approach might be complex, but with modern software, the MI procedure is straightforward to implement and is considered best practice for rigorous analysis of even modest amounts of missing data [2].

**Pattern-Mixture Sensitivity Analysis under MNAR**

We agree that assuming Missing At Random is untestable, so we have planned a pattern-mixture analysis using the δ-adjustment method to assess the robustness of our findings to potential Missing Not At Random (MNAR) mechanisms [1]. We have clarified this plan in the manuscript. In brief, this analysis will deliberately introduce a fixed offset (δ) to the imputed outcome values for participants with missing data, to simulate a scenario where those with missing outcomes have systematically different pain scores than those observed [2]. This approach is aligned with recommendations by Cro et al. (2020) for conducting controlled MI-based sensitivity analyses in confirmatory trials [2].

**Choice of δ (delta) parameters:** We will explore a range of plausible δ values for both endpoints (VAS and SCAI) to represent potential worst-case (or best-case) departures from the MAR assumption. For the VAS (pain score), which is measured on a 0-10 scale (or 0 -100 mm on the VAS ruler with slider), we consider shifts up to about +1.0 point as realistic since a 1-point difference on the VAS is the minimal clinically important difference we used in our sample size calculation, and thus represents a meaningful change in pain perception. For the SCAI (Skin Conductance Algesimeter Index, measured in µS/s), we will use a similar approach. Because the SCAI is on a different scale, we will apply δ values corresponding to roughly 10% increases in the pain-induced conductance level. In practical terms, if the average SCAI reading is X µS/s, we might add a δ of about +0.05 - 0.1 µS/s to missing observations, which represents a moderate worsening in pain response based on device readings. We believe these δ values (for VAS and SCAI, respectively) are large enough to reflect meaningful deviations (e.g., a patient with missing data potentially had notably more pain), yet still within a plausible range informed by clinical judgment.

**Tipping point” analysis**: After applying these δ shifts, we will re-run the primary between-group comparisons. We will identify the tipping point at which the conclusion changes. Specifically, the value of δ at which the p-value for the group difference crosses the 0.05 threshold. For example, if the primary analysis shows a significant benefit of the microneedle patch, we will increase δ stepwise (making the missing outcomes gradually worse in the microneedle group or better in the control group, as a conservative scenario) until the difference is no longer statistically significant (p ≥ 0.05). This δ value represents the tipping point where our result would lose significance. Conversely, if an outcome were non-significant in the primary analysis, we would examine if a favourable δ shift could tip it to significance. We will report these findings to demonstrate how robust the treatment effect is to potential MNAR bias. This form of patterned missing data analysis is essentially a stress test for our conclusions. If the results remain significant even with reasonably large δ offsets, readers can be confident in the robustness of our trial findings. However, if the results are sensitive to a very small δ, that will be transparently acknowledged in our manuscript reporting the trial results.

By providing the δ values examined and the tipping point where the inference changes, we adhere to recommended practice. This will directly address the concern about whether our findings could be dependent on the assumption of MAR. We will clarify in the manuscript that our primary analysis assumes MAR (with MI), but we have a planned δ-adjustment analysis to evaluate how much missing-not-at-random deviation it would take to alter the study conclusions.

**ii) Manuscript changes (In Methodology, Statistical Analysis Plan Subsection)**

“Specifically, we will employ multiple imputation by chained equations (MICE) with a predictive mean matching algorithm to impute missing outcome data [26]. We will generate 20 multiply-imputed datasets (increasing the number of imputations if the precision is unsatisfactory) and include baseline VAS and SCAI scores as auxiliary variables in the imputation model. This approach ensures that all randomized participants are analysed and that the imputation draws on relevant pre-pain measurements to make the MAR assumption as plausible as possible [27].

To assess departures from MAR (i.e., Missing Not At Random (MNAR)), we will perform pattern-mixture models with delta adjustment and a tipping-point analysis over clinically-plausible offset [27]. We will explore a range of δ values for the pain outcomes (e.g., up to ±1.0 VAS point and up to ± 10% in SCAI units) to represent plausible worse-outcome scenarios for those with missing data. The results will be re-evaluated under each δ and we will identify the tipping point at which the statistical significance of the between-group difference changes (from p<0.05 to p≥0.05, or vice versa). This will illustrate how robust the primary outcome is to departures from the MAR assumption.” (**Pages 28-29, Lines 552-566**)

**iii) References Used in Responses**

1. Cro S, Morris TP, Kenward MG, Carpenter JR. Sensitivity analysis for clinical trials with missing continuous outcome data using controlled multiple imputation: A practical guide. Stat Med. 2020;39(21):2815-2842. doi: 10.1002/sim.8569. **[reference 26 in the manuscript text]**

2. White IR, Royston P, Wood AM. Multiple imputation using chained equations: Issues and guidance for practice. Stat Med. 2011;30(4):377-99. doi: 10.1002/sim.4067. **[reference 25 in the manuscript text].**

_____________________________________________________________________

**10) Reviewer’s Comment 6e:** Excluding influential observations conflicts with ITT; instead present them as sensitivity analyses.

**i) Author’s Response**

Thank you for the clarification. We agree that removing observations would conflict with the intention-to-treat (ITT) principle if used for the primary analysis. Our primary analysis remains ITT with all randomised participants analysed as allocated.

As noted in the revised text, the analysis that omits influential observations is only for sensitivity analysis. We will fit the primary model after temporarily omitting observations identified as influential by standard diagnostics and compare the estimates with those from the full ITT dataset.

**ii) Manuscript changes (In Methodology, Trial Status Subsection)**

“Besides, in a separate sensitivity analysis, we will also fit the primary model after excluding observations identified as influential by standard diagnostics (Cook’s distance, DFFITS, and DFBETAS) and compare the estimates with those obtained using the full dataset.” (**Pages 30-31, Lines 606-609**)

_____________________________________________________________________

**11) Reviewer’s Comment 6f:** The Abstract/Methods (lines 40-44) mention 142 participants = 71/arm, whereas the power section (lines 170-178) recalculates 72 per arm; 144 total. Kindly harmonise these figures throughout the manuscript.

**i) Author’s Response**

Thank you for pointing this out. We have harmonised the Abstract to match the sample size section. It now states 72 participants per arm, 144 in total.

**ii) Manuscript changes (In Abstract)**

“Twenty (single-group LEMAP recipients) and 144 adult patients (72 per group; randomised to either LEMAP (intervention) or 5% EMLA patch (control) applied on antecubital fossa, …” (**Page 2, Lines 35-36**)

__________________________________________________________________

**12) Reviewer’s Comment 7:** Recruitment traffic-light plan is clear; nonetheless, the red-light threshold (< 9 participants / month) may prematurely terminate the trial if clinic flow fluctuates, consider adding a buffer period.

**i) Author’s Response**

Thank you for this suggestion. We have added a buffer period so that the red threshold will not be triggered on a transient dip in monthly participant recruitment. **Red** now requires sustained under-recruitment for three months (less than an average of nine participants per month for three months) before any decision to terminate the trial.

**ii) Manuscript changes (In Methodology, Participant Recruitment and Retention Strategy and Recruitment Monitoring Subsection)**

**“Red**: Fewer than nine participants are recruited and randomised per month, with an attrition rate below 10 percent, or the three-month rolling average remains below nine participants despite mitigation.” (**Page 26, Lines 495-497**)

_____________________________________________________________________

**13) Reviewer’s Comment 8:** Stopping criteria are mentioned for Phase I PK, but Phase II lacks explicit adverse-event grading and reporting timelines. A brief statement (e.g., “All AEs graded per CTCAE v5.0 and reported within 24 h to the DSMB”) would close that gap.

**i) Author’s Response**

We thank the reviewer for highlighting this. We agree clarity of stopping criteria is essential for Phase II. The protocol already specifies AE grading (CTCAE v5.0 and FDA toxicity grading) and reporting timelines (SAEs within 24 h; Grade ≥3 AEs within 5 business days) and defines trial halting (stopping) criteria. However, these were presented in the general Safety Assessment Section rather than explicitly within the Phase II section. We have now inserted an explicit Phase II safety statement and clarified that the trial halting (stopping) criteria apply to both phases. These changes harmonise the details for Phase I and Phase II while keeping the procedures unchanged.

**ii) Manuscript changes (In Methodology, Subsection Safety Assessment)**

“The following definitions, grading framework, reporting timelines, and oversight apply to both Phase I and Phase II.” (**Page 24, Lines 436-437**)

“The study halting criteria above apply to both Phase I and Phase II.” (**Page 25, Line 477**)

_____________________________________________________________________

**14) Reviewer’s Comment 9:** Data-availability detail – The Harvard Dataverse statement is welcome, but it should pre-specify

a) the file types to be shared (e.g., raw and processed VAS/SCAI data, PK curves, blank CRFs),

b) a variable dictionary/codebook, and

c) the intended licence (e.g., CC-BY 4.0).

**i) Author’s Response**

We thank the reviewer for such an insightful comment. We have revised the Data availability section to meet all three points. It now pre-specifies the files to be shared and the licence. The Harvard Dataverse deposit will include raw and processed VAS and SCAI data, de-identified analysis datasets, PK concentration-time data and derived outputs, blank CRFs, screening and randomisation logs, the protocol deviation log, and a complete variable dictionary and codebook. All files will be released under the Creative Commons Attribution 4.0 International licence.

**ii) Manuscript changes (In Methodology, Ethical Issue and Dissemination of Results Subsection)**

“The trial data will be shared on Harvard Dataverse (https://dataverse.harvard.edu) to ensure the transparent dissemination of study findings and adherence to recommendations made by leading medical journal editors for the future publication of this research. The deposit will include raw and processed VAS and SCAI outcome data, de-identified analysis datasets, PK concentration–time data and derived outputs, blank CRFs, screening and randomisation logs, protocol deviation log, and a complete variable dictionary and codebook. All shared files will be released under a Creative Commons Attribution 4.0 International licence.” (**Page 28, Lines 537-543**)

_____________________________________________________________________

**15) Reviewer’s Comment 10:** Competing-interest declaration – The methods section cites a patent application for the microneedle patch (UKM.IKB.800-4/1/5849, filed 16 Apr 2024), yet the “Competing interests” field states “None declared.” Please clarify whether any authors are inventors, hold a licence agreement, or have a financial stake, and update the declaration accordingly.

**i) Author’s Response**

Thank you. We have corrected the Conflict of Interests declaration to address this point (see below).

**ii) Manuscript changes (In Conflict of Interest Section)**

“AAH, DCF, MAM, MRB and PCO are named inventors on a patent application for LEMAP (UKM.IKB.800-4/1/5849), owned by UKM. There is no licence or option agreement in place. No author has any personal financial interest, royalties, equity, or paid role related to this technology. The remaining authors declare no competing interests.” (**Page 36, Lines 749-752**)

_____________________________________________________________________

**B) Reviewer 2: Point-by-Point Responses**

**1) Reviewer’s Comment 1:** The power analysis for the phase II study was properly justified. However, authors must explain or provide justification or references regarding why they considered a 1-point VAS difference between the intervention group as the minimum detectable difference (MTD), as sample size is largely influenced in trial studies by (MDD). Note is MDD, not MTD.

**i) Author’s Response**

We thank the reviewer for this helpful comment. We agree that all pain outcomes should be expressed on the 0-100 mm VAS scale. We have corrected the units in the Phase II sample size paragraph and now state a 10 mm target difference with SD 20 mm, which corresponds to the same standardized effect size (Cohen’s d = 0.5) used previously. A 10 mm difference is consistent with the lower end of published minimal clinically important differences (MCIDs) for acute pain and has been repeatedly supported in emergency and perioperative studies [1-5]. Todd et al. reported about 13 mm as the smallest important change adults can perceive on a 100 mm VAS [1], with similar findings from Gallagher et al [2]. Kelly reported an MCID of 9 mm VAS in an emergency setting [3], and a postoperative study by Myles et al. triangulated an MCID of approximately 10 mm [4]. A systematic review found threshold-based MCIDs with a median of 10 mm VAS score across acute pain studies, based on the threshold method for absolute VAS change [5].

On this basis, 10 mm is a reasonable and conservative value to power a confirmatory trial. Re-powering to ≥13-15 mm would assume a larger effect than is necessary and could reduce the required sample size while increasing the risk of type II error if the true effect is closer to 10 mm. We therefore retain 10 mm (SD 20 mm) and have clarified the units in the manuscript with citations to the MCID literature.

We also have removed minimum detectable difference (MDD) and replaced it with MCID to standardise the terminology.

**ii) Manuscript changes (In Methodology, Sample Size Calculation Subsection, Under Phase II: Efficacy Study Sub-subsection)**

“We consider a 10-mm point VAS difference on the 0-100 mm VAS scale between the intervention group as the MCID for acute procedural pain, based on the threshold approach reported in Olsen and colleagues in their systematic review [12] and to reduce the probability of type II error if the true treatment effect is actually closer to this conservative choice of MCID.” (**Page 13, Lines 173-176**)

**iii) References Used in Responses**

1) Todd KH, Funk KG, Funk JP, Bonacci R. Clinical significance of reported changes in pain severity. Ann Emerg Med. 1996;27(4):485-9.

2) Gallagher EJ, Liebman M, Bijur PE. Prospective validation of clinically important changes in pain severity measured on a visual analog scale. Ann Emerg Med. 2001;38(6):633-8.

3) Kelly AM. The minimum clinically significant difference in visual analogue scale pain score does not differ with severity of pain. Emerg Med J. 2001;18(3):205-7.

4) Myles PS, Myles DB, Galagher W, et al. Minimal clinically important difference for three quality of recovery scales after general anaesthesia. Br J Anaesth. 2017;118(3):424-9

5) Olsen MF, Bjerre E, Hansen MD, et al. Pain relief that matters to patients: Systematic review of MCID for acute pain measured on VAS/NRS. BMC Med. 2017;15:35. (**reference 12 in the main manuscript text**)

_____________________________________________________________________

**2) Reviewer’s Comment 2:** For missing observations, the authors indicated that they will use the multiple imputation method to fill in the missing data, assuming the missing at random (MAR) mechanism. Since data are yet to be collected, it will be more prudent to include in the proposal other missingness mechanisms and how they will be addressed if they occur, as it is not guaranteed that the missingness mechanism will follow MAR. Why not MNAR, MCAR etc

**i) Author’s Response**

Thank you for your comment. Our primary analytical plan for addressing missing data (multiple imputation under a Missing At Random (MAR) assumption) is standard for clinical trials. Missing Completely At Random (MCAR) is generally implausible in trials with treatment-related withdrawal, as noted by Cro and colleagues [1]. We already prespecified MNAR based on pattern-mixture / δ-adjustment and tipping-point analyses to assess departures from MAR, which addresses the reviewer’s request to consider mechanisms beyond MAR.

**ii) Manuscript changes (In Methodology, Under Statistical Analysis Plan Subsection)**

“For missing observations, we will use the multiple imputation method to fill in the missing data, assuming the missing at random (MAR) mechanism if the proportion of missing data per variable is more than 5% [25]. (**Page 28, Lines 550-552**)

To assess departures from MAR (i.e. Missing Not At Random (MNAR)) we will perform pattern-mixture models with delta adjustment and a tipping-point analysis over clinically plausible offset [27].” (**Page 29, Lines 559-561**)

**iii) References Used In Response**

1. Cro S, Morris TP, Kenward MG, Carpenter JR. Sensitivity analysis for clinical trials with missing continuous outcome data using controlled multiple imputation: A practical guide. Stat Med. 2020;39(21):2815-2842. doi: 10.1002/sim.8569 (This is referenced as reference no 27 in the main manuscript text)

_____________________________________________________________________

**3) Reviewer’s Comment 3:** Assuming MLR is not appropriate, include a non-parametric regression modelling alternative to MLR in the proposal, similar to what you did for the t-test by proposing the Mann-Whitney test

**i) Author’s Response**

Thank you for the suggestion. Our primary analysis uses covariate-adjusted multiple linear regression, which is appropriate for our continuous endpoints. We will check residuals, functional form, and variance, and apply transformation where indicated. We appreciate the concern about assumptions, although at present there is no evidence that MLR will be unsuitable.

To address the possibility of unresolved violations, we have pre-specified two alternatives. If diagnostics indicate important issues that are not corrected by transformation, we will fit quantile regression at the median using the same covariates and report an adjusted median difference with bootstrap confidence intervals. We will also fit a generalised additive model (GAM) with penalised splines for continuous covariates while retaining a parametric treatment term. This wording has been added to the Statistical Analysis Plan.

**ii) Manuscript changes (In Methodology, Under Statistical Analysis Plan Subsection)**

“If the MLR model diagnostics indicate important assumption violations that are unresolved by transformation, we will proceed with two pre-specified alternatives. First, we will fit quantile regression at the median using the same covariates and report an adjusted median difference with bootstrap confidence intervals (CIs). Second, we will also fit a generalised additive model (GAM) with penalised splines for continuous covariates while retaining a parametric treatment term.” (**Page 30, Lines 593-598**)

_____________________________________________________________________

**4) Reviewer’s Comment 4:** Line 521, kindly delete R2, is R-squared not R2. Kindly use proper equation editor to write it out well

**i) Author’s Response**

Thank you for the correction. We have removed “R2” and formatted it as R² using the equation editor in the document on **Page 30, Line 585**.

_____________________________________________________________________
